# The model structure of the hammerhead ribozyme formed by RNAs of reciprocal chirality

**DOI:** 10.1042/BSR20203424

**Published:** 2021-01-08

**Authors:** Eliza Wyszko, Mariusz Popenda, Dorota Gudanis, Joanna Sarzyńska, Agnieszka Belter, Patrick Perrigue, Paweł Skowronek, Katarzyna Rolle, Jan Barciszewski

**Affiliations:** 1 Institute of Bioorganic Chemistry of the Polish Academy of Sciences, Noskowskiego 12, 61-704 Poznań, Poland; 2 NanoBiomedical Center of the Adam Mickiewicz University, Wszechnicy Piastowskiej 3, 61-614 Poznań, Poland; 3 Faculty of Chemistry of the Adam Mickiewicz University, Uniwersytetu Poznańskiego 8, 61-614 Poznań, Poland

**Keywords:** catalytic RNA, enantiomeric ribozymes, irregular Watson-Crick base pairs, mirror-image nucleic acids, RNA modelling, RNA-zyme

## Abstract

RNA-based tools are frequently used to modulate gene expression in living cells. However, the stability and effectiveness of such RNA-based tools is limited by cellular nuclease activity. One way to increase RNA’s resistance to nucleases is to replace its D-ribose backbone with L-ribose isomers. This modification changes chirality of an entire RNA molecule to L-form giving it more chance of survival when introduced into cells. Recently, we have described the activity of left-handed hammerhead ribozyme (L-Rz, L-HH) that can specifically hydrolyse RNA with the opposite chirality at a predetermined location. To understand the structural background of the RNA specific cleavage in a heterochiral complex, we used circular dichroism (CD) and nuclear magnetic resonance (NMR) spectroscopy as well as performed molecular modelling and dynamics simulations of homo- and heterochiral RNA complexes. The active ribozyme-target heterochiral complex showed a mixed chirality as well as low field imino proton NMR signals. We modelled the 3D structures of the oligoribonucleotides with their ribozyme counterparts of reciprocal chirality. L- or D-ribozyme formed a stable, homochiral helix 2, and two short double heterochiral helixes 1 and 3 of D- or L-RNA strand thorough irregular Watson–Crick base pairs. The formation of the heterochiral complexes is supported by the result of simulation molecular dynamics. These new observations suggest that L-catalytic nucleic acids can be used as tools in translational biology and diagnostics.

## Introduction

A fundamental feature of the proteins and nucleic acids is their homochirality. These natural biopolymers are composed of only one optically active ‘isomer’: L- amino acids or D-nucleotides. Although the origin of homochirality in biological macromolecules is not fully understood, it is assumed to be essential to ensure the high specificity and fidelity of the recognition mechanism between them. The stereospecificity of enzyme (protein) or ribozyme (nucleic acids) catalysed reactions is the foundation of the homochiral world.

Changing chirality is a strategy to overcome the low chemical stability of the phosphodiester bond of D-RNA in order to build more effective RNA-based tools. It is known that mirror-image ribonucleic acids consisting of L-ribose instead of D-ribose are resistant to hydrolysis with ribonucleases. Also L-DNA, where D-deoxyribose is substituted with L-deoxyribose, are resistant to nucleases [1,2]. Short interference RNAs (siRNAs) and catalytic RNAs (ribozymes) are widely used for modulation of gene expression [3], and it seems that a stable L-counterpart could be more suitable and attractive for their effectiveness in therapeutic applications [4]. It is generally accepted that the biggest advantage of nucleic acids for various applications is due to the propensity for complementary recognition of relevant nucleic acid strands by Watson–Crick base ‘pairing’: but these interactions require the substrate of the same chirality. This means that D-RNA or D-DNA can form base pairs with another D-RNA or D-DNA. However, 30 years ago, L-DNA was proposed for use in antisense technologies, but stable duplexes of L/D-RNA with Watson–Crick base pairs have not been demonstrated [2,5,6]. Other studies have indicated that RNA heterochiral complexes are also not formed [7,8]. Similarly, ‘L-oligonucleotides’ composed of all four canonical nucleotides did not form a heterochiral helix with D-RNA or D-DNA [9]. On the other hand, heterochiral complexes between homopurine and homopyrimidine strands have been proposed, without the identification of an interaction mode between nucleic acid bases. Formation of non-canonical heterochiral pairs between L-(dAp)_5_dA and D-poly(rU) have been suggested, but no hybridization of L-dU oligomer and D-poly(dA) was observed [10]. Also no complementarity has been detected between of L- and D-DNA with RNA containing all four base residues [9]. A few years ago it has been demonstrated that the mirror image RNA aptamer (L-RNA) binds to TAR RNA (D-RNA) and inhibits the formation of the TAR-tat complex critical to HIV-1 replication [11]. Recently, it has been shown *in vitro* that selected L-DNA aptamers bind to a D-RNA target [12], but Watson–Crick base pairs within that complex have been excluded [11,12].

The intense interest in enantiomeric nucleic acids observed in the last 40 years has dampened recently because the finding that L- and D- oligonucleotides do not form a regular double helix with Watson–Crick base pairs [13].

Recently, for the first time, some of us have shown that mirror image hammerhead ribozymes (L-ribozyme) and L-DNAzyme cleave the complementary D-RNA substrate of reciprocal chirality at the predetermined site [14,15]. On this basis, one can conclude that the enantiomeric nucleic acids could be used for regulation of their biological activity, and L-RNA could make valuable tools for potential therapeutic applications [15]. Here, we present new experimental results and extensive 3D molecular modelling data, that allow us to conclude that the interactions of the L-ribozyme with D-RNA target are possible. The results are based on the formation of irregular base-pairs with Watson–Crick type hydrogen bonding, that ensures the cleavage capacity and specificity of the L-ribozyme. The specific homo/heterochiral complex formation of the mixed chirality between D-substrate and L-ribozyme take place due to the strong homochiral structure of the central part of the ribozyme (helix 2), which induces and stabilizes conformational changes (χ, P angles and helical parameters) in the substrate strand, and facilitates its binding to the ribozyme. Our model also provides an explanation of why a complex of two linear complementary oligonucleotides with similar length, and reverse chirality cannot be formed.

## Materials and methods

### Oligonucleotides synthesis

Chemically synthesized RNA oligonucleotides were from IBA (Germany) or ChemGenes (U.S.A.). The 14-nt synthetic D- and L-RNA oligomers with the sequence 5′CUUCAAGUCCGCCA3′ were labelled with fluorescein at the 5′ end. The D-HH (D-Rz), L-HH (L-Rz) hammerhead ribozymes were 33-nt long with the sequence 5′UGGCGCUGAUGAGGCCGAAAGGCCGAAACUUGA3′. Nucleotides of L33 at positions 1-6 and 29-33 for parallel complex were methylated at 2′OH. D-anti HH Ribozyme 5′UGUCAGCUGAUGAGGCCGAAAGGCCGAACCAGCGG3′ was synthesized and labelled with FAM.

### RNA hydrolysis with ribozymes *in vitro*

The activity of the D- and the L-hammerhead ribozymes with labelled L- and D-RNA targets were measured in 10 μl reaction volumes containing 50 mM Tris-HCl at pH 7.5 at 37°C. The RNA substrate and L- or D-Rz ribozymes were denatured for 2 min at 73°C and cooled to 25°C at a rate of 1°C/min. The reactions were done at 0 and 25 mM MgCl_2_ concentrations. Hydrolysis products were separated with 20% polyacrylamide gel electrophoresis (PAGE) in the presence of 7 M urea in 0.09 M Tris-borate buffer at pH 8.3. The gels were exposed to a Fuji Film FLA 5100 phosphoimager using the manufacturer’s (Fuji) BCIP/NBT Liquid Substrate System (Sigma), and bands were quantified using the ImageQuant software from Molecular Dynamics.

### CD spectroscopy

CD spectra were recorded on a JASCO J-815 spectropolarimeter. The L- and D-oligonucleotides were dissolved in 50 mM NaCl, 20 mM Tris-HCl pH 7.5 buffer to achieve a concentration of 3.0 μM. All samples were denatured at 90°C for 5 min and then slowly cooled to room temperature before data collection [17]. The spectra were recorded in triplicate at 25°C in the 205–320 nm wavelength range with a 1 nm data interval. The buffer spectrum was subtracted from the sample spectra.

### NMR spectroscopy

All NMR spectra were acquired on a Bruker AVANCE III 700 MHz spectrometer, equipped with a QCI CryoProbe. D-RNA, L-Rz and their equimolar amounts (3 μM) complex 3 were dissolved in buffer 150 mM NaCl, 50 mM Tris-HCl pH 7.5 with 10 mM MgCl_2_. Equimolar amounts D-RNA and L-Rz (L33) was dissolved in 150 mM NaCl and 10 mM sodium phosphate pH 6.8. The solvent was H_2_O/D_2_O (9:1, v/v). The samples were annealed by heating at 90°C for 5 min and then slowly cooled to room temperature. The 3 mm thin wall tubes were used with a final sample volume of 200 μl. The water signal was suppressed by excitation sculpting with a gradient pulse. Spectra were processed and prepared with TopSpin 3.2 Bruker Software.

### Structure modelling and molecular simulations

The 3D structures of homochiral and heterochiral complexes of RNA hammerhead (Rz) ribozyme (33-mer) and RNA oligonucleotide (14-mer) were constructed based on the 3D crystal structure of the catalytic center of hammerhead ribozyme (3ZP8) with RNA Composer [16]. The 14 nt RNA substrate strand was joined to the ribozyme 3′-end through the GGG linker (17). This allowed building a model of the cis-acting ribozyme. RNA secondary structure predictions were performed through the Vienna RNAfold server (http://rna.tbi.univie.ac.at/cgi-bin/RNAfold.cgi). We incorporated the three-way junction motif covering the catalytic center of the ribozyme (PDB: 3ZP8). RNAfold generates up to ten structure models and we selected only the model with the lowest total energy. Visualizations of the molecular models were done in PyMOL (http://www.pymol.org). Finally, the linker was removed, and the structure was stored. The heterochiral model L/D we got from D/D structure with the X-Plor program ver. 2.21 [18]. The parameters for force field CHARMM (dna_rna_allatom.param) were extended with parameters for L-RNA nucleotides. Parameters for L-RNA differ from D-RNA only for the torsion angles nucleosides and impropers for that enforce ribose chirality. Parameters kchimpr differ only in the sign [18–21]. Furthermore, 1000 step minimalization was done using conjugate Powel Method, and restrained hydrogen bonds and planarity restrains for Watson–Crick base pairs.

Geometry of the D/D and D/L duplex models were of heterochiral complex. Correctness of the centre chirality was evaluated by a data processing validated with RCSB MAXIT (MAcromolecularEXchange Input Tool), covering geometry of covalent bond and chirality centres. The two other models L/L and L/D were obtained by changes of values of the Z axis from D/D and D/L, respectively.

The fours starting structures (models) of DL, LD, LL and DD were built using SYBYL and UCSF Chimera [21,22]. In the double-stranded segments, Watson–Crick base pairs were held with proper alignment and distance for each hydrogen bond. All these models was subjected to MD simulations.

MD simulations were performed with the AMBER18 package [23]. We used the *tleap* module of Amber 18 to prepare the topologies and coordinates for simulations. We utilized the AMBER ff99bsc0χ_OL3_ force field for D-RNA and its modified version for L-RNA [24,25]. The modification of ff99bsc0χ_OL3_ to account for L-ribonucleotides involved corrections of dihedral angles (γ and χ). Each of the structures was solvated with TIP3P water molecules in a truncated octahedral box with minimal distance of 10 Å between solute and the box border [26]. The systems were neutralized with Na^+^ cations and NaCl excess salt was added to obtain the concentration of 150 mM by using Joung and Cheatham ion parameters [27]. The solvated systems were minimized and equilibrated. The minimization involved three series of 200 steps with steepest descent followed by 300 steps with the conjugate gradient method, first with position restraints of 25.5 kcal/mol Å^2^ applied to solute atoms and finally without any restraints. Then, at the heating stage, the temperature was gradually raised to 300 K within 80 ps followed by 20 ps of equilibration while solute atoms restrained with harmonic potential of 15 kcal/(mol·Å^2^). Next, the restraints were gradually released in four rounds (10, 5, 1 and 0.5 kcal/mol Å^2^) of 100 ps each. After that, two sets of MD simulations were carried out: one set of entirely unrestrained simulations and a second set with small harmonic potential of 0.5 kcal/ mol·Å^2^ applied to base heavy atoms of residues involved in Watson–Crick hydrogen bonding between heterochiral strands, to preserve these bonds and to allow the sugar-phospate backbone to adjust to it. The long-range electrostatics interactions were calculated using the particle mesh Ewald method, periodic boundary conditions, and the non-bonded cut-off set to 9 Å. The covalent bonds were constrained using SHAKE, and the integration time step was set to 2 fs. The Langevin thermostat with collision frequency 2.0 ps ^−1^ was used to control the temperature and the Berendsen barostat was used to enforce a constant pressure simulation. MD simulations were performed with the *pmemd* module of AMBER 18 on CUDA Device (Ge Force RTX2080). The data generated from MD were analysed using the CPPTRAJ module of Amber Tools [26].

## Results

**Figure 1:**
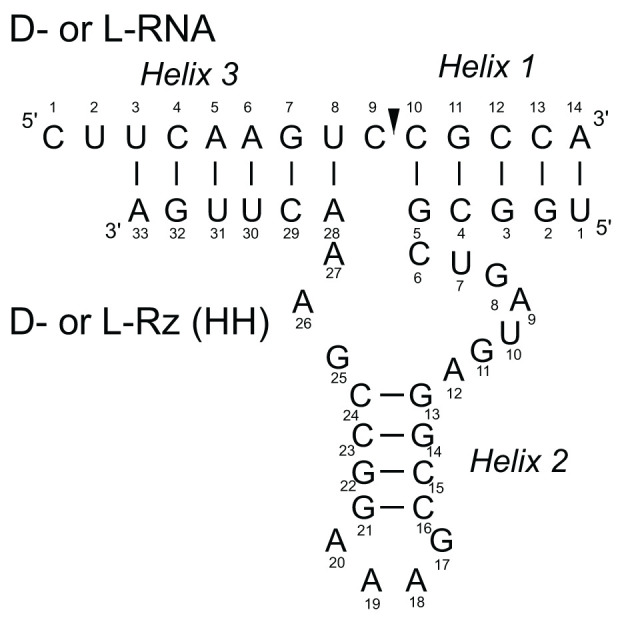
The secondary structural model of a L/D-hammerhead ribozyme (Rz) complexed with an L/D-RNA substrate containing GUC↓N, the predetermined cleavage site for hammerhead ribozymes The arrow identifies specific cleavage site for all ribozymes. Each heterochiral catalytic complex contains the classical Watson–Crick homochiral base pairs forming stem core of ribozyme helix 2, (stem 2), which induce and stabilize heterochiral Watson–Crick like base pairs with substrate helices 1 and 3.

Recently, we have shown that L-hammerhead ribozyme (L-Rz, L-HH) hydrolysed a D-RNA target [15]. The chemically synthesized 33-nucleotide L-ribozyme (longer or bigger partner) cleaved the 14 nucleotides D-RNA substrate (shorter or smaller partner) at the predetermined location of GUC↓N (Figure 1). The model of mixed chirality between L-hammerhead ribozyme with D-RNA substrate is based on nucleotide sequence complementarity. Two short heterochiral helixes (helices 1 and 3) are formed between flanking parts of L-ribozyme with the substrate, but helix 2 of the catalytic L-RNA is always homochiral and stable.

**Figure 2:**
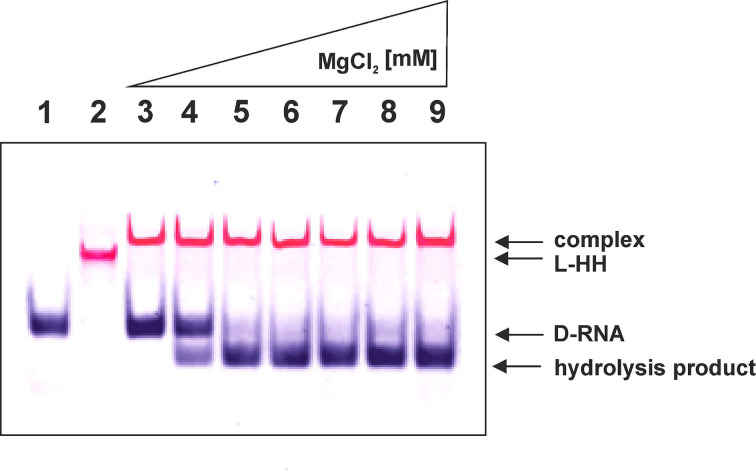
Visualization of intermediates and D-RNA hydrolysis with L-hammerhead ribozyme (L-HH) D-RNA substrate of 14 nucleotide long (0.25 μM) labelled with FAM was incubated with 2.5 μM L-HH ribozyme labelled with cy5 and 4 μM cold L-HH at 70°C during 2 min, then mixture was fast cooled down, then reaction was incubated during 3h at 37°C in presence of increasing concentration of MgCl_2_ (0.5, 1, 5, 10, 15, 20, 25 mM, lanes 3, 4, 5, 6, 7, 8, 9, respectively) in binding buffer (50 mM Tris-HCl pH 7.5, 160 mM KCl, 10 mM spermidine). Analysis was done on 15% native polyacrylamide gel with 2.5% glycerol. Lane: 1- D-RNA; 2 – L-HH ribozyme.

**Figure 3:**
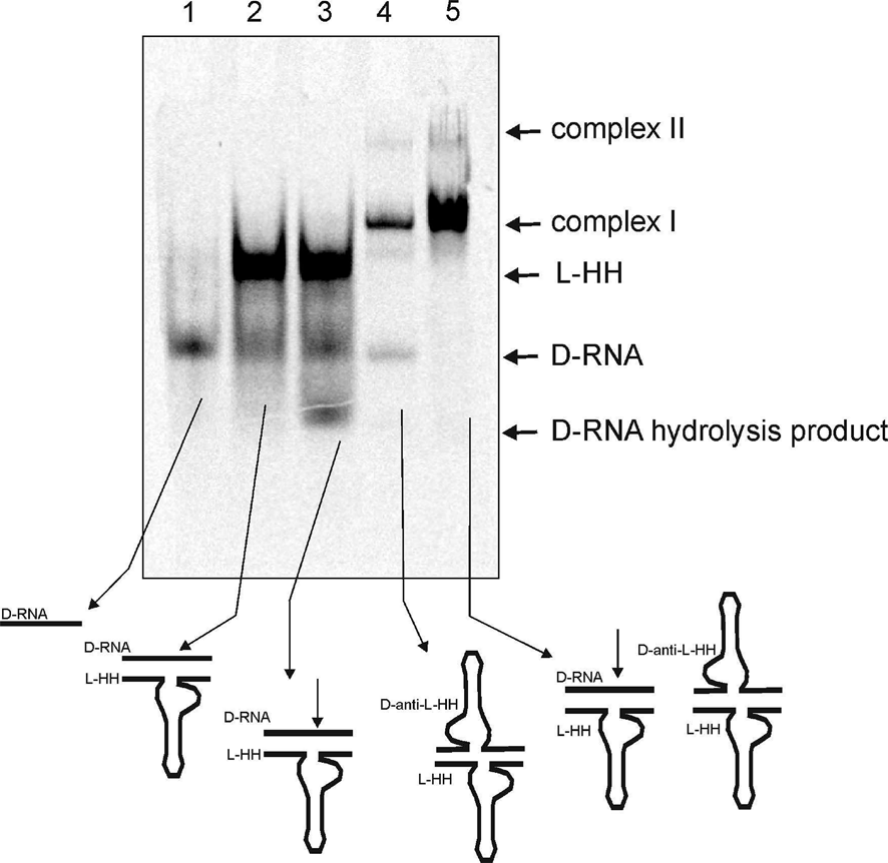
Analysis of complex formation of heterochiral RNAs 1. D-RNA target incubated in binding buffer containing 25 mM MgCl_2_ (see Figure 2), 2. D-RNA target incubated in binding buffer with L-HH ribozyme, 3. D-RNA and L-HH ribozyme incubated in binding buffer containing 25 mM MgCl_2_, 4. D-RNA, L-Rz ribozyme and anti D-Rz ribozyme in binding buffer, 5. D-RNA, L-Rz ribozyme and anti D-Rz ribozyme incubated in binding buffer containing 25 mM MgCl_2_. Arrows shows cleavage products and complex formation position.

To understand a mechanism of the active complex formation between D-RNA substrate and L-RNA ribozyme, we checked its migration on a native polyacrylamide gel. Using specifically colour-labelled reagents, we were able to show that D-RNA substrate forms the complex with L-ribozyme (L-HH) and migrates slowly (Figure 2). One can see, that in the presence of magnesium and 20 fold excess, L-hammerhead (HH) ribozyme specifically cleaved D-RNA target [15], and the expected hydrolysis products were observed (Figure 2). This observation was confirmed with results on Figure 3. One should notice that specific hydrolysis takes place in the presence of magnesium ions (Figure 3, Lane 3). Interestingly, no specific cleavage was observed when L-HH ribozyme was complexed with D-anti-HH-ribozyme even in the presence of MgCl_2_ (Figure 3). One should notice that both molecules have the strong secondary structure, particularly helix 2 and the same size.

**Figure 4:**
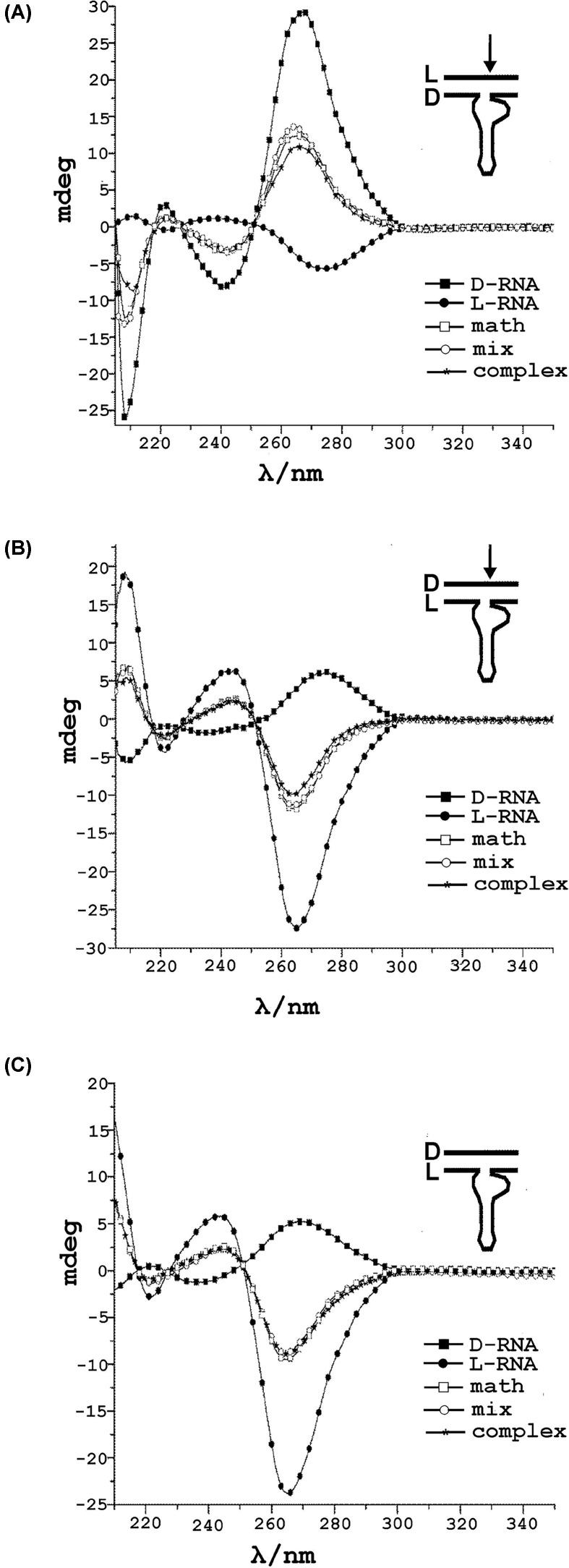
Circular dichroism (CD) spectra of heterochiral complexes of L- and D-hammerhead ribozyme with D- or L-RNA targets in 20 mM Tris pH 7.5 and 50 mM NaCl at room temperature CD spectra are plotted as ellipticity (millidegrees) versus wavelength. Smooth curves represent the average of three separated scans at 1 nm sampling interval from 210 to 350 nm. (**A**) CD spectra of L-RNA target in complex with D-RNA (hammerhead ribozyme). (**B**) D-RNA target with L-RNA (hammerhead ribozyme). (**C**) control experiment with the use of D-RNA (5′GGCGACCGACUGU3′) not fully complementary to L-RNA (hammerhead ribozyme). There are differences in the Cotton effect of the complexes. Black squares correspond to D- or L-RNA (ribozyme), (33-mer); black circles refer to L- or D-RNA target (14-mer), open squares represent mathematical sum of CD spectra of hammerhead ribozyme and RNA target, open circles correspond to a mixture of D-or L-hammerhead ribozyme with D-or L-RNA target without incubation step. Black stars show a complex of D- or L-RNA (ribozyme) with D- or L-RNA target preincubated at 90°C and slowly renatured overnight.

To get a deeper insight into the conformation of the heterochiral complexes, we measured the CD spectra of L/D-ribozymes with L/D-RNA substrates (Figure 4). The L- or D-ribozymes are characterized by negative or positive CD band at 263 nm, respectively. The CD spectra of the homochiral structures for left-handed L-RNA and right-handed D-RNA substrates adopt negative or positive Cotton effect, respectively. That CD plot for L-HH is a mirror image of the CD plot obtained for the structure of the D-hammerhead ribozyme (D-HH). For the heterochiral complex of a ribozyme with RNA substrate, a difference between the Cotton effect of the renatured complex, the mixture of the ribozyme, and the substrate and their mathematical sum was detected (Figure 4A,B). The observed final Cotton effect for heterochiral complexes is reduced in comparison to the sum of individual components and the mixture of these components.

Figure 4A shows a right-handed complex because of the presence of D-RNA, for example, D-hammerhead ribozyme and Figure 4B presents a left-handed complex due to L-RNA, for example, L-hammerhead ribozyme. The important contribution of the sequence complementarity between ribozyme and the target for heterochiral complex formation was seen on Figure 4C. The lack of specific base pairing (Watson–Crick base pairs) between the target D-RNA and the L-RNA (hammerhead ribozyme) did not lead to the complex formation and therefore specific hydrolysis was excluded (Figure 4C).

**Figure 5:**
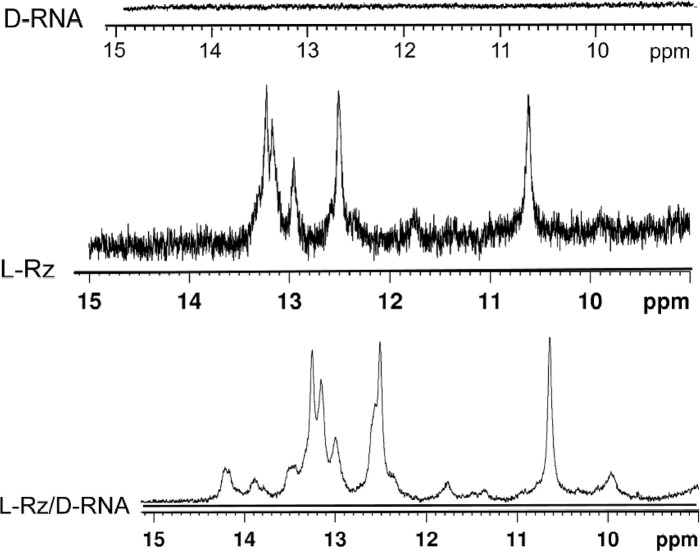
The imino region of ^1^H NMR spectra of: D-RNA target, L-Rz ribozyme alone and D-RNA/L-Rz complex with 150 mM sodium chloride, 10 mM Tris-HCl buffer and 0.1 mM EDTA at 25°C

To confirm the formation of heterochiral complexes and evaluate the nature of hydrogen bonding interactions in these complexes, we recorded the ^1^H NMR spectra. They show spectra of single-stranded D-RNA or L-RNA (Figure 4) without imino proton signals. This indicated the lack of intrinsic secondary structure for the substrate strands. In contrast, several imino proton signals in the 10-14 ppm region were observed for the L-ribozyme, in general, in agreement with their secondary structures predicted by the RNA structure software [21,22]. For the heterochiral complex D-RNA/L-Rz, new imino-proton signals appeared in the range of 13.5-14.5 ppm (Figure 5). This is clearly demonstrated by the formation of Watson–Crick hydrogen bonds which stabilize the complex. To confirm the specific complex formation, we recordered ^1^H NMR spectrum of putative heterochiral complex of L-Rz/D-RNA designed in such a way that helices 1 and 3 should form the parallel duplex (Supplementary Figure S1). For the complex with parallel stems new imino-proton signals in the region of 13–14.5 ppm were also observed: however, their pattern and intensity were different than those obtained for anti-parallel heterochiral catalytic complex (Supplementary Figure S2). It suggests that the nature of the hydrogen bond interactions is different for the parallel and anti-parallel complex.

Having experimental data on the specific hydrolysis of RNA with catalytic nucleic acids (zymes) in a heterochiral manner [15] as well as biophysical data (this paper), we calculated the 3D structures of the active heterochiral complexes. Because of the experimentally observed high specificity of D-RNA cleavage, it is obvious that the L-catalytic nucleic acids and the D-RNA substrate form a double strand with Watson–Crick hydrogen bonding network. It is required for the formation of the active site of ribozyme and the specific cleavage of the substrate at the GUC target [28,29].

**Figure 6:**
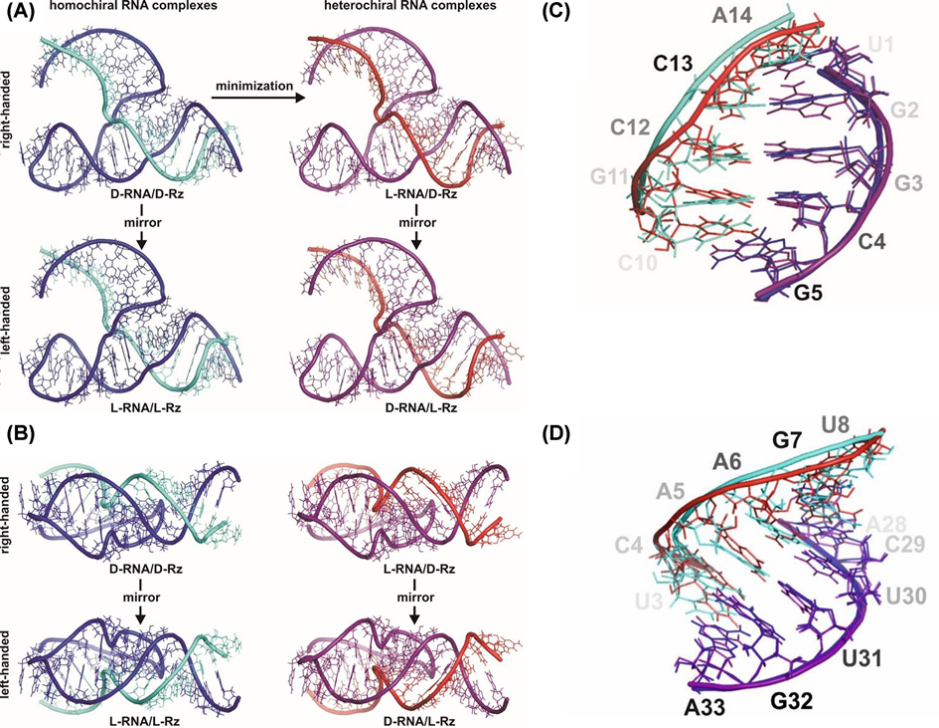
The 3D structure model of homochiral and heterochiral complexes of RNA hammerhead (Rz) ribozyme (34-mer) with RNA oligonucleotide (14-mer) as well as their fragments, modeled with Composer [18] and X-plor [19], respectively (**A**) Transformation of right handed homochiral to heterochiral RNA complex by energy minimalization. Left handed complexes were obtained by mirror image. (**B**) Perpendicular view of four RNA complexes generated in A: D-RNA/D-Rz; L-RNA/D-Rz; L-RNA/L-Rz and D-RNA/L-Rz. (**C**) Overlapping of the 5′ end of D-hammerhead ribozyme in complex with D- RNA or L-RNA targets. The pentanucleotide C_10_GCCA_14_ sequence of D-RNA (green) or L-RNA target (red) at the 3′end forms Watson–Crick base pairs with U_1_GGCG_5_ residues at the 5′ end of D-RNA hammerhead ribozyme. (**D**) Overlapping of the 3′ end of D-hammerhead ribozyme in complex with 5′ end of D- RNA (green) or L-RNA (red) target. Watson–Crick interactions between U_3_CAAGU_8_ of D- or L-RNA target sequence at the 5′end with hexanucleotide of A_28_CUUGA_33_ at the 3′ end of D-RNA hammerhead ribozyme are visible.

To understand how this complex is self-assembled, we developed a new approach to find the possible configuration of L-nucleosides. We started with the right-handed homochiral complex of D-RNA/D-ribozyme, called the D/D RNA model (Figure 6A). The model of the 3D structure of homochiral right-handed complex of D-RNA target with D-Rz (ribozyme) was calculated on the basis of the crystallographic data (3ZP8) using an automated 3D structure modelling algorithm, Composer, invented by one of us [16]. The secondary structure of the hammerhead ribozyme input data was the same as in [17]. The D/D RNA model was transformed into heterochiral complex L-RNA/D-Rz (Figure 6A) by energy minimization with the Charm Field Force [19–21] using X-plor programme [18].

The parameters of the complex, such as the bond lengths, angles between covalent bonds, electrostatic charges, and Van den Waals radii were the same for L-nucleotides and for their D enancjomers [21,22], except for the chiral centers and torsial angles, which have the opposite sign. These parameters allow a configuration change from D-RNA to L-RNA during energy minimalization (Figure 6A). The complexes of homochiral L-RNA/L-Rz complex and heterochiral D-RNA/L-Rz complex were obtained by mirror operations (Figure 6A,B). The sign of the atomic *Z-*coordinates of the right-handed (D-RNA/D-Rz) homochiral complex multiplied by -1 leads into the left-handed homochiral L-RNA/L-Rz complex (Figure 6A). The mirror image of L-RNA/D-Rz leads to D-RNA/L-Rz (Figure 6A,B).

Comparison of both the overall homochiral model complexes L/L and D/D (Figure 6) for heavy atoms (1000 atoms) showed Root Mean Square Deviation (RMSD) of 10.038 Å, but the RMSD for the RNA substrate and ribozyme were 5.520 Å (289 atoms) and 9.321 Å (711 atoms), respectively. On the other hand, a comparison of the heterochiral complex L/D with its homochiral counterpart D/D carried out with the Composer, showed a RMSD of 0.894 Å. Interestingly, the RMSD for a D substrate (289 atoms) was 1.484 Å, but it was 0.446 Å for L-ribozyme (711 atoms).

**Figure 7:**
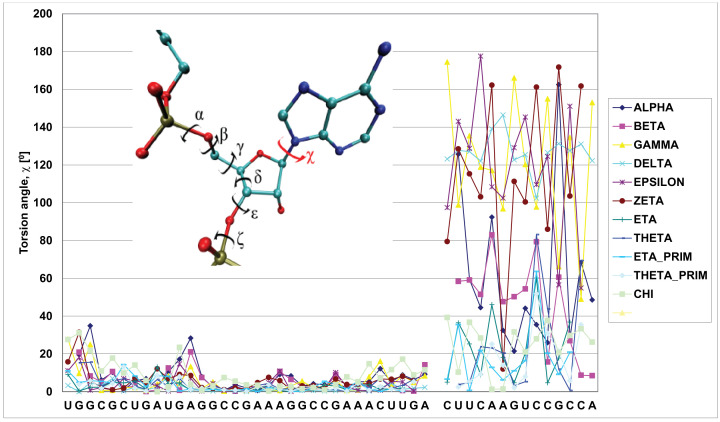
Differences of values of angles for all nucleotides in D- and L- hammerhead ribozyme and D and L-RNA target oligonucleotides

**Figure 8:**
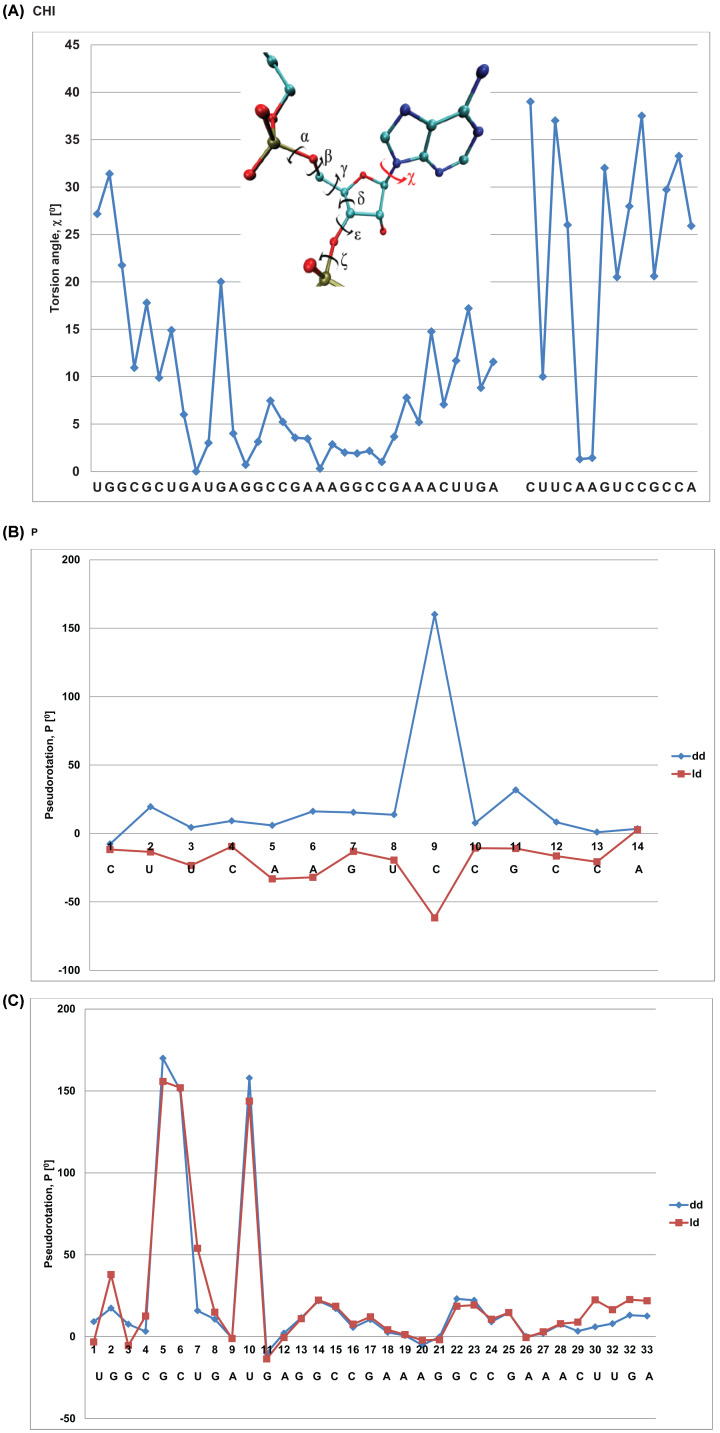
Differences of values of (Chi) torsion angle (**A**) and pseudorotation (P) angle (**B and C**) for homo- and heterochiral complexes; (**B**) RNA substrate, (**C**) - L-ribozyme dd – homochiral complex; ld – heterochiral complex

**Figure 9:**
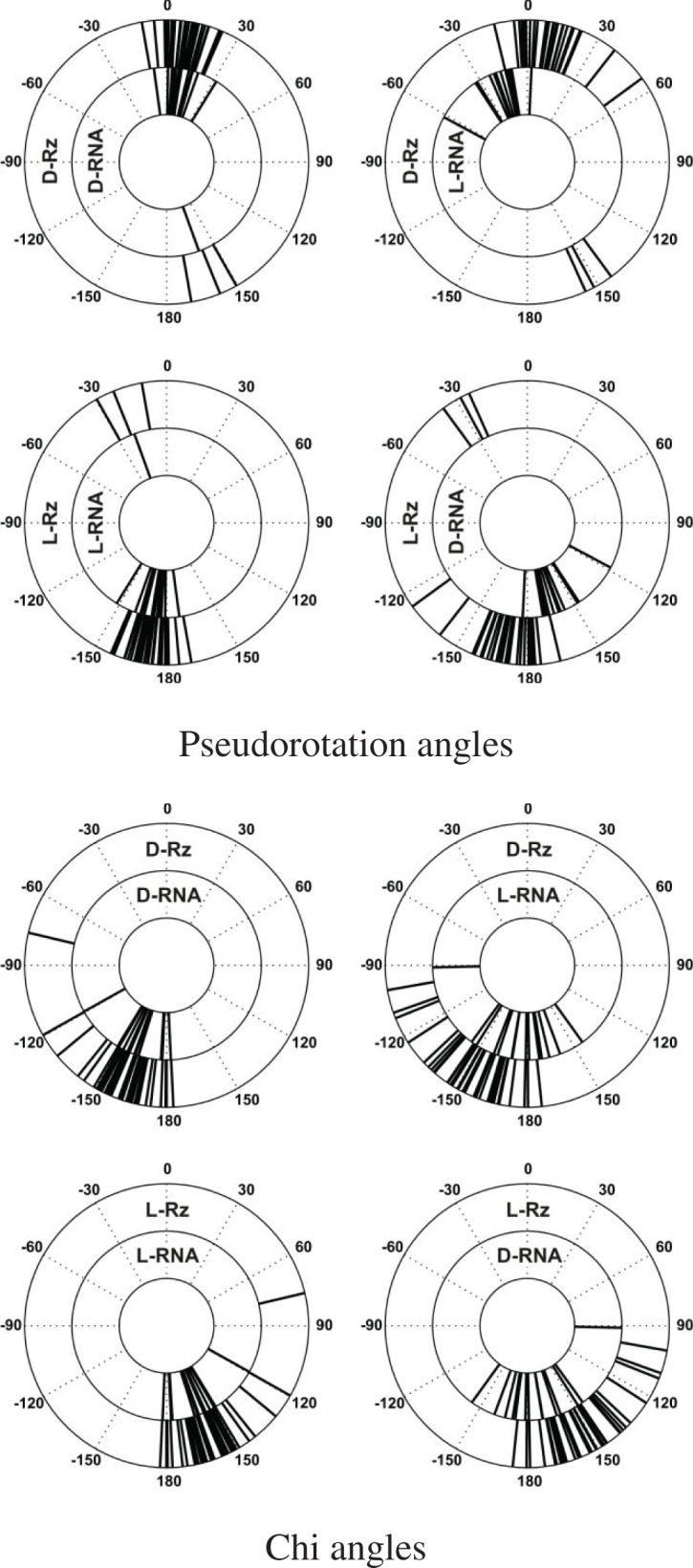
Circular diagram of pseudo rotation torsion and chi angle values for the target and hammerhead ribozyme molecules, in all homo- and heterochiral duplexes

Different views of all four model complexes showed their structural similarities (Figure 6A,B). The most important part of the heterochiral complex models were interactions between RNAs of reverse chirality, for example, the double helixes 1 and 3 [H1, H3]. For H3 U_3_CAAGU_8_ of D- or L-target and A_28_CUUGA_33_ of D- or L-Rz (HH), one can clearly see that Watson-Crick type base pairs within the heterochiral complex were very similar to those of the homochiral complex with some irregularities (Figure 6D). RMSD for the heptanucleotide heterochiral duplex of 383 atoms is 1.279 Å. Detailed comparison of parts of the target RNA and the ribozyme showed RMSD of 0.950 and 0.408 Å, respectively. The same concerns the helix 1 (H1) of the other end of the complex (Figure 6C). The RMSD of heterochiral double helix of residues 10–14 of target RNA with residues of 1–5 of the ribozyme is 1.438 Å (for 322 atoms), but RMSD of each part of the complex target RNA and the ribozyme, is 1.014 and 0.617 Å, respectively. The small alterations observed in nucleoside conformation of the double-stranded RNA (Figure 6C,D) are due to the variations in the backbond angles (Figure 7). To get insight on the structure of the heterochiral complex, we analysed differences in values of all angles characteristic for a nucleotide conformation (Figure 7 and Supplementary Figures S3–6). One can see only minor variations in the angles of the L-hammerhead ribozyme structure (Figure 7, left sequence). On the other hand, differences in the structure of the target D-RNA model are larger (Figure 7, right sequence). The values of Chi orientation of base about glycosidic bond (Chi angle) values in heterochiral complex model differ, although all nucleosides exist in anti conformation (Figures 7 and 8A). One should also notice that the phase angle of the pseudorotation (P) values are similar in the homochiral complex (Figure 8B,C) and nucleoside 9 (cleavage site) shows the S conformation (C2′endo) [30–32]. In the heterochiral complex, this nucleotide occurs in the N conformation (C3′endo) (Figure 8B). A summary of pseudo rotation (P) and Chi angles changes (Figure 9) clearly showed the full symmetry between D-Rz/D-RNA and L-Rz/L-RNA homochiral, as well as D-Rz/L-RNA and L-Rz/D-RNA heterochiral complex models. In the next step, we calculated RMSD of ribose-phosphate backbone to evaluate the stability of two homochiral D/D and L/L (Supplementary Figure S7) and heterochiral D/L and L/D complexes without any restrictions (free) (Supplementary Figure S8). The RMSD for whole homochiral complexes show small fluctuations; formation of the D/D and L/L homochiral complexes is effected by classical Watson-Crick base pairs. The same concerned the active centre and all three helices. From analysis of the heterochiral D/L and L/D complexes one can see similar trajectories for helix (stem) 2 that is actually homochiral, and helixes (stems) 1 and 3 that are heterochiral. The last ones can be formed by slightly modified Watson–Crick base pairing. Trajectories for the complexes with constraints on the heavy atoms seem to be similar (Supplementary Figure S9). The observed changes are correlated with the structure of the ribozyme and show small conformational changes within the helixes 1 and 3 as well as in the active site. One should keep in mind that stem 2 (helix 2), due to its homochirality contains 4 G-C base pairs and is very stable. It is responsible for the active site formation. It seems that pairing of the substrate with flanking parts of the ribozyme is required for specificity of ribozyme cleavage and stabilization of the complex.

**Figure 10:**
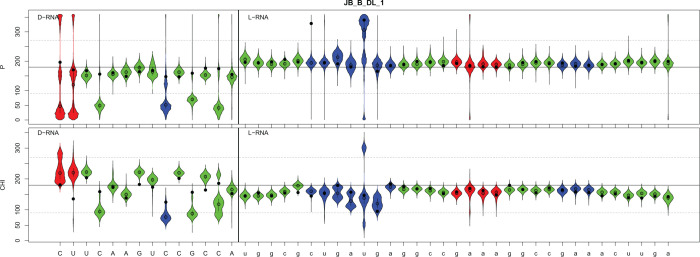
The violin plot analysis of pseudorotation phase (P) and chi angle values of D-RNA/L-RNA ribozyme complex. Red – single strand nucleosides, green – paired nucleosides, blue – single stranded catalytic centre D-DNA - capital, L-RNA – small letters.

Next, we checked the phase pseudorotation (P) and the chi angle values of the calculated heterochiral complex. The violin plot analysis of the molecular dynamics simulation of the D-RNA/L-Rz complex with positional restraints shows that all nucleotides of the L-Rz adopt C2′-endo sugar pucker (S-type) with pseudororation phase values (P) of approximately 180^0^ (Figure 10). This supports the stable conformation of left-handed ribozyme. The conformation of nucleotides of D-RNA substrate show different, mixed P values. These changes occur regularly. At the 3′-end of D-RNA nucleotides U8, C10, C12 and U14 show 2′ endo (S-type) sugar puckers, but C4, C9, C11 and C13 show 3′-endo (N-type). All the bases of the L-ribozyme are in the anti-conformation with chi angle values of approximately 160^0^. Each of the second nucleotide of D-RNA substrate occurs in anti- and syn-conformation (Figure 10). These results suggest that the solution structure of the D-RNA/L-ribozyme heterochiral complex is similar to that of Z-RNA [33,34].

## Discussion

The molecular structures can be chiral, and have mirror-image isomers of the opposite chirality. These isomers are called enantiomers or optical isomers, because they rotate plane-polarized light either to the right (D) or to the left (L). The building blocks of the life are homochiral, which means that naturally occurring amino acids, protein, sugars, and nucleic acids exist as only one chiral form. If these building blocks had mixed chirality, molecular chaos would ensue, and life would not be possible.

In principle, mirror-image or looking-glass versions of chiral molecules should work in the same way as original ones, but they are resistant to attack by viruses or enzymes that have not evolved in a glass-looking world. That suggestion opens the door to a new dimension of mirror-image molecular biology with broad applications in diagnostics and therapeutics. During last 40 years there have been many studies showing the formation of complexes of two strands of opposite chriality; however, the conclusions drawn from their structure are not clear [13].

In this work, we were interested in the structure of mirror image catalytic RNAs (L-RNA) and its interaction with RNA of reciprocal chirality (D-RNA). We concentrated on chemistry of the ribozyme because a specific RNA (heterochiral or homochiral) hydrolysis is based on two important premises: (i) the tertiary structure of the active center should allow the substrate to adopt a precise conformation and formation of a transient active structure, and (ii) the active site of both the homochiral and heterochiral complexes should be very similar to perform the same specific catalysis.

The D-hammerhead ribozyme activity against various D-RNAs (D-targets) has been studied recently [14,15,30–32]. The crystal structure of the D-hammerhead ribozyme (3ZP8) shows that the catalytic core adopts a well-defined structure that places the nucleophile 2′-oxygen of the target nucleotide in position for an in-line attack [28,29]. Recently, we have shown that the ribozyme specificity in the homochiral complexes (D/D and L/L) as well heterochiral (L/D and D/L) where the substrate and ribozyme have different structure (length size) is totally preserved and occurs at the predetermined GUC↓N cleavage site of the substrate [14,15]. Therefore, it is obvious that the catalytic site of the homochiral and heterochiral complexes should follow similar folding patterns. Similarly to homochiral complex, formation of the avctive heterochiral counterpart can take place only in the presence of magnesium ions (Figures 2 and 3).

Binding of D-RNA to L-Rz was observed on the gel (Figure 3 lane 2), but only after addition of Mg^+2^ ions, specific hydrolysis takes place. One can also see that L-HHRz binds to D-anti-HHRz (Figure 3, lane 4) by Mg^+2^ was not enough for hydrolysis. It seems that very stable structure of both molecules did not allow them to aquire of the active conformation.

In the CD spectra recorded for the annealed ribozyme-substrate complexes, the change in amplitude at 265 nm relative to the mathematical sum of both strands reflects conformational variation resulting from the formation of antiparallel heterochiral complexes (Figure 4A,B).

Additionally, we performed a control experiment in which we have used D-RNA substrate strand with the sequence 5′GGCGACCGACUGU3′ not fully complementary to L-Rz (ribozyme). After binding to the substrate to L-Rz, no change of the intensity of the CD signals was detected indicating that in this case the heterochiral complex did not form (Figure 4C).

Analysis of NMR spectra fully confirmed the formation of heterochiral L-Rz/D-RNA complex. New imino signals in the range of 13.5–14.5 ppm were observed in NMR spectrum when L-Rz and L-RNA/D-RNA was annealed (Figure 5).

We have also consider parallel complex formation of D-RNA target with L-HH ribozyme. However NMR spectra of such complex (Supplementary Figure S1) did not show down-field imine protons (Supplementary Figure S2), and therefore did not support a parallel helices 1 and 3.

Our 3D structure of the heterochiral models evidently shows that Watson-Crick base pairs between the ribozyme and substrate can be form although their geometry are slightly different (Figure 6C,D).

It is interesting that using standard, well-known modeling tools and simple mathematical operation one can get antiparallel heterochiral Watson–Crick base pairs, that have geometry that is very similar with the canonical Watson–Crick base pairs (Figure 6C,D). As one can see within the complex of L-hammerhead ribozyme with D-RNA, the most important helices are H1 and H3 as well as the catalytic center (Figure 1). Helix 2 (stem 2) of the D- or L-hammerhead ribozymes are always homochiral and very stable due to 4 consecutive G-C pairs [31]. Helix H2 in L-RNA is also left handed (homochiral), when in the complex with the D-RNA target. It is known that almost all nucleotides of the L-ribozyme show S configuration of ribose and anti-conformation of glycosidic bond (chi angle). Only 3 nucleotides have the N conformation in the single-stranded region, which is typical for D-RNA. Formation of Watson-Crick base pairs within the helices 1 and 3 show S configuration for L-nucleotides of ribozyme and also for D-nucleotides of RNA-target [32]. That changes of N to S of nucleotide conformation in the RNA target was induced by the stable structure of L-ribozyme (helix 2). Such a conformation is not surprising; an one can observe that for example in Z-RNA [12,17,34]. Cytidines in Z-RNA shows S configuration of sugar in nucleotides and anti conformation of nucleotides. On the other hand guanine shows N configuration of sugar and syn conformation of nucleotides [28]. One should mention here that in the heteroduplex of DNA–RNA, there are also different sugar puckers. The sugar puckers were predominantly either 3′ endo (A-RNA or DNA) or 2′ endo (B-DNA). In the heteroduplex DNA–RNA the distance between the neighboring phosphorus (P) atoms and orientation of the P relative to the sugar are different. They are 5.9 Å for C3′ exo (N) and 7.0 Å for C2′ endo (S) conformations, respectively.

To obtain deeper structural insight into the heterochiral complexes, we studied the molecular dynamics simulations of the complexes. These results suggest that the Watson-Crick like type pairing formed by strands of reversed chirality in helixes 1 and 3, are induced and stabilized by the homochiral helix 2 of the ribozyme. Such interactions maintain the active center of the catalytic RNA. Pairing between flanking L-nucleosides of ribozyme and D-RNA substrate is possible due to changes within sugar puckers and syn-anti conformation changes, which occur regularly every second nucleoside (Figure 10). Our results on heterochiral complex formation between oligo RNA of reversed chirality with different lengths confirm earlier observations on the formation heterochiral complexes [10]. In our case L-ribozyme (33 nt) binds and cleaves D-RNA of 14 nt, but previous work showed heterochiral complex of L-(dA_5_)A with D-poly (U), [10]. That observation provides arguments the against current understanding that heterochiral specific Watson–Crick base pairs are not possible [8].

## Supplementary Material

Supplementary Figures S1-S9

## Abbreviations

CD: circular dichroism
L-Rz, L-HH: left-handed hammerhead ribozyme
NMR: nuclear magnetic resonance
siRNA: short interference RNA
