# Supplementary material for: The model structure of the hammerhead ribozyme formed by RNAs of reciprocal chirality
Source: Biosci Rep. 2021 Jan 8;41(1):BSR20203424. doi: 10.1042/BSR20203424 (PMC7796190; doi:10.1042/BSR20203424)
Supplement: Supplementary Figures S1-S9 [file BSR-2020-3424_supp.pdf]

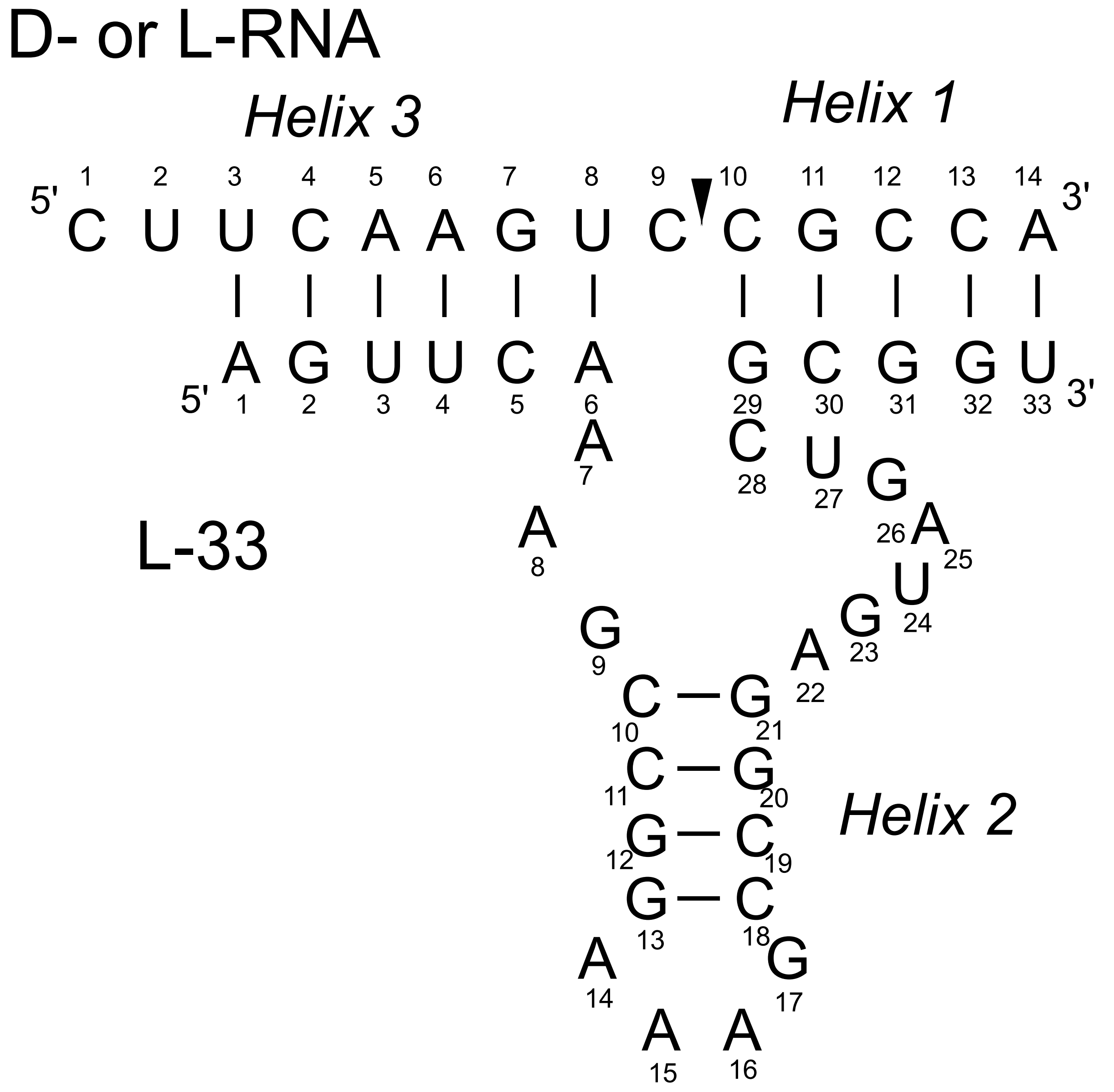

D- or L-RNA
Helix 3
Helix 1
5' C U U C A A G U C C G C C A 3'
5' A G U U C A G C G G U 3'
L-33
Helix 2

Supplementary Figure 1. The secondary structure model of L-Rz/D-RNA putative parallel complex. The both nucleic acid strands run parallel from 5’ to 3’ end. Residues of 1-6 and 29-33 are 2’-O-CH_3 analogues.

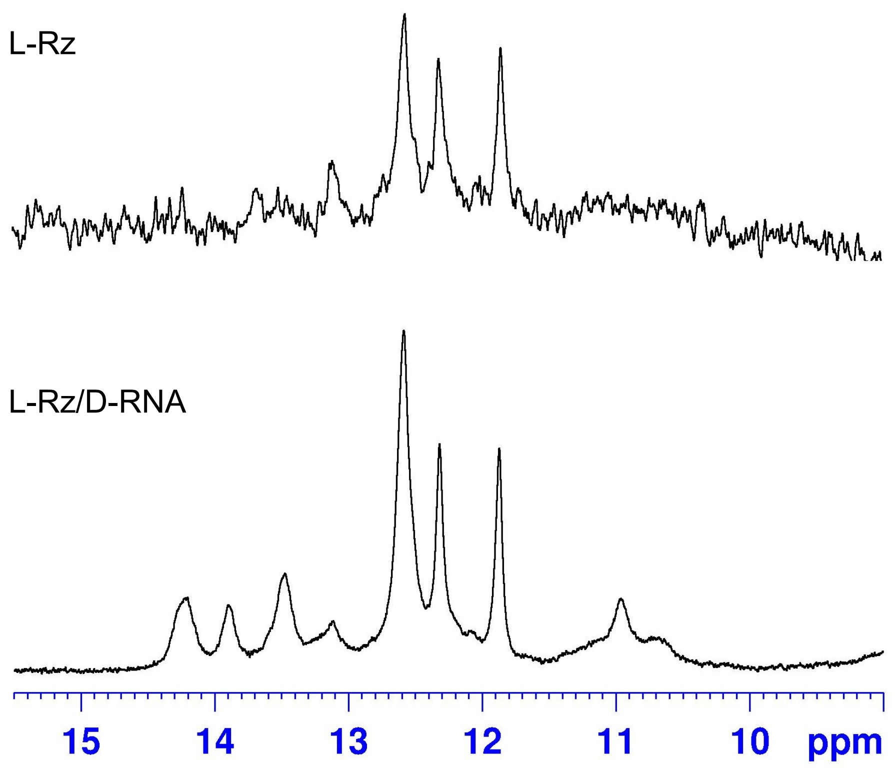

L-Rz
L-Rz/D-RNA
15
14
13
12
11
10
ppm

Supplementary Figure 2. ^{1}H NMR spectrumof the putative parallel complex of L-Rz and D-RNA.D-RNA target, L-Rz alone and L-Rz/D-RNA parallel complex with 150 mM sodium chloride, 10 mM phosphate sodium buffer pH 6.8 and 0.1 mM EDTA.

Supplementary Fig. 3.

D-target / D-ribozyme

				alpha	beta	gamma	delta	v0	v1	v2	v3	v4	P	fi	chi	epsi	zeta
1	A	1	C c	....	....	21.366	90.226	17.627	-35.063	38.302	-29.235	7.446	-7.734	38.653	-176.509	-111.026	-63.033
2	A	2	U U	-46.787	153.260	54.614	82.240	-0.583	-22.696	36.023	-37.437	23.921	19.493	38.213	-160.081	-141.569	-58.962
3	A	3	U U	-68.174	176.487	47.443	86.061	9.744	-30.318	38.280	-33.747	15.188	4.307	38.389	-156.899	-154.743	-64.293
4	A	4	C C	-63.419	-168.774	35.807	83.824	6.584	-28.629	38.614	-35.899	18.514	9.178	39.114	-152.727	-154.947	-77.075
5	A	5	A A	-56.952	-179.178	39.213	85.106	8.801	-29.940	38.553	-34.581	16.317	5.850	38.755	-148.829	-154.722	-71.277
6	A	6	A A	-64.714	169.374	58.132	82.239	1.740	-24.926	37.312	-37.383	22.470	16.120	38.839	-160.765	-155.226	-74.293
7	A	7	G G	-69.731	179.326	54.871	83.841	2.219	-24.542	36.234	-36.022	21.332	15.341	37.573	-155.639	-156.622	-59.029
8	A	8	U U	-72.061	-166.985	42.143	86.780	3.167	-23.695	34.008	-33.268	19.007	13.647	34.996	-149.772	-125.579	-80.833
9	A	9	C C	-49.994	179.794	44.694	141.940	-23.128	35.618	-33.805	21.594	0.744	159.986	35.978	-119.223	-110.108	87.206
10	A	10	C C	78.990	-141.368	-75.633	85.054	7.454	-28.578	37.724	-34.512	17.094	7.640	38.062	-162.605	-125.778	-78.259
11	A	11	G G	-80.927	147.103	83.618	81.370	-8.832	-15.334	32.154	-38.145	29.542	31.728	37.803	177.494	-154.920	-64.279
12	A	12	C C	-74.374	-163.385	40.445	84.984	7.077	-28.397	37.737	-34.751	17.497	8.218	38.129	-149.326	-124.839	-82.385
13	A	13	C C	-88.733	115.061	106.604	86.786	12.038	-31.873	38.565	-32.679	13.085	0.895	38.569	-177.337		
14	A	14	A A	-74.069	-162.507	45.198	86.712	10.270	-30.383	37.838	-33.021	14.428	3.340	37.902	-162.724	-70.740	-30.014
15	B	1	U u	....	....	57.917	86.080	6.279	-27.184	36.563	-34.007	17.538	9.129	37.032	-163.467	-160.732	-57.819
16	B	2	G G	-69.475	-162.123	37.766	81.471	0.855	-24.546	37.548	-38.127	23.452	17.384	39.345	-154.141	-133.265	-79.203
17	B	3	G G	-92.980	121.417	111.047	86.319	7.235	-27.612	36.362	-33.277	16.470	7.584	36.683	-179.582	-145.097	-67.763
18	B	4	C C	-68.609	-173.708	44.451	86.649	10.354	-30.554	38.064	-33.169	14.438	3.273	38.126	-156.942	-150.889	-74.422
19	B	5	G G	-64.480	-172.070	61.889	146.574	-18.233	34.282	-36.392	26.971	-5.696	169.951	36.959	-129.649	-165.887	-108.997
20	B	6	C C	-67.396	-175.810	43.612	138.742	-30.431	40.415	-34.669	18.156	7.501	150.572	39.805	-119.443	-88.370	154.595
21	B	7	U U	-80.029	111.864	76.116	84.754	1.832	-23.626	35.183	-35.167	21.026	15.846	36.573	-152.242	-147.487	-47.501
22	B	8	G G	152.385	149.229	60.567	85.197	5.263	-26.505	36.476	-34.529	18.496	10.722	37.124	179.958	-135.643	-68.930
23	B	9	A A	-59.584	169.384	42.132	85.583	13.886	-34.393	40.748	-33.792	12.663	-0.833	40.752	-160.222	-114.470	-69.838
24	B	10	U U	-7.844	-137.265	41.712	139.671	-24.288	36.031	-33.499	20.534	2.147	157.867	36.163	-141.336	-80.687	59.751
25	B	11	G G	-128.453	-165.461	52.811	93.611	17.874	-33.483	35.575	-26.357	5.514	-10.091	36.134	-76.689	-155.810	99.913
26	B	12	A A	-48.652	140.079	38.378	87.198	10.988	-30.667	37.635	-32.401	13.583	2.140	37.662	177.297	-127.770	-64.205
27	B	13	G G	-62.861	166.158	54.154	82.511	4.992	-27.893	38.933	-37.129	20.265	11.560	39.739	-173.510	-158.088	-71.101
28	B	14	G G	-62.527	-172.737	49.002	80.561	-2.284	-22.141	36.749	-39.107	26.046	21.826	39.587	-167.878	-149.165	-71.564
29	B	15	C C	-83.511	170.006	71.328	81.767	0.992	-24.562	37.456	-37.915	23.253	17.168	39.202	-165.707	-158.311	-71.753
30	B	16	C C	-66.411	-166.741	38.710	85.536	8.909	-29.894	38.384	-34.359	16.092	5.631	38.570	-151.287	-153.911	-59.607
31	B	17	G G	-69.482	-175.912	45.580	83.599	5.690	-27.922	38.303	-36.122	19.198	10.433	38.947	-155.190	-142.321	-63.266
32	B	18	A A	144.611	154.520	62.997	88.616	10.229	-29.227	36.033	-31.244	13.336	2.645	36.072	-171.537	-141.592	-67.299
33	B	19	A A	-73.744	161.422	66.845	89.134	11.486	-30.159	36.325	-30.801	12.281	0.736	36.328	-164.032	-157.899	-51.759
34	B	20	A A	-78.586	-164.728	45.923	91.301	14.997	-32.116	36.084	-28.543	8.669	-5.094	36.227	-144.394	-137.327	-80.967
35	B	21	G G	120.757	-120.675	-160.908	88.132	12.608	-31.902	38.009	-31.870	12.251	-0.191	38.010	-177.614	-145.813	-64.435
36	B	22	G G	-66.079	-178.400	53.428	81.092	-3.145	-20.995	35.720	-38.552	26.245	23.124	38.841	-167.010	-155.551	-61.680
37	B	23	C C	-69.058	-175.907	48.491	82.190	-2.432	-20.969	35.001	-37.440	25.101	22.219	37.809	-152.722	-153.336	-73.972
38	B	24	C C	101.884	-156.775	-129.683	84.721	6.539	-28.161	37.891	-35.195	18.080	9.050	38.369	-164.723	-145.073	-53.673
39	B	25	G G	-67.769	163.185	64.541	83.999	2.636	-24.815	36.308	-35.847	20.940	14.710	37.538	-168.405	-167.313	-108.742
40	B	26	A A	-57.211	-166.720	46.860	88.982	11.980	-30.670	36.650	-30.862	12.008	0.111	36.650	-161.581	-146.149	-59.852
41	B	27	A A	-67.364	174.504	48.661	86.072	11.404	-31.763	38.959	-33.478	13.997	2.058	38.985	-156.068	-147.217	-75.234
42	B	28	A A	-61.972	156.179	62.698	85.665	7.444	-28.391	37.364	-34.174	16.911	7.554	37.691	-160.529	-140.536	-76.901
43	B	29	C C	-64.925	-179.689	40.847	85.806	10.506	-31.173	38.862	-33.903	14.850	3.385	38.930	-155.144	-157.121	-68.728
44	B	30	U U	-63.304	-173.959	41.453	85.990	8.513	-29.272	37.742	-33.906	16.078	5.995	37.949	-149.143	-154.912	-77.649
45	B	31	U U	-60.871	178.141	45.900	85.006	7.255	-28.461	37.695	-34.623	17.292	7.948	38.061	-150.496	-151.162	-77.442
46	B	32	G G	-73.614	165.784	65.777	84.089	3.736	-25.845	36.853	-35.802	20.215	13.120	37.841	-161.707	-157.906	-56.055
47	B	33	A A	-73.534	-160.870	37.994	84.918	3.987	-25.264	35.690	-34.500	19.274	12.585	36.568	-150.788	....	....

Supplementary Figure 3. The values of all angles characteristic for nucleotide conformation for the D-target/D-ribozyme.

Supplementary Fig. 4

D-target / L-ribozyme

				alpha	beta	gamma	delta	v0	v1	v2	v3	v4	P	fi	chi	epsi	zeta
1	A	1	C c	....	....	164.222	146.659	-19.375	35.116	-36.601	26.458	-4.659	168.277	37.381	-144.241	13.607	142.497
2	A	2	U U	-78.887	-94.873	-153.391	150.023	-23.339	40.503	-41.712	29.198	-3.783	166.483	42.900	170.421	-75.401	-172.580
3	A	3	U U	8.817	-117.388	177.122	146.878	-31.210	45.305	-41.734	24.706	3.889	156.557	45.489	-166.375	-76.543	179.578
4	A	4	C C	18.954	-139.762	-154.618	154.137	-21.027	40.115	-43.093	31.851	-6.946	170.437	43.701	-178.880	-27.506	-179.805
5	A	5	A A	-35.403	-97.865	-156.265	135.915	-32.079	40.331	-33.142	15.635	10.124	146.741	39.635	147.522	-96.868	-126.533
6	A	6	A A	32.259	-121.755	-154.915	131.336	-27.558	35.260	-29.300	14.385	8.057	147.935	34.574	159.336	52.774	86.271
7	A	7	G G	91.145	130.433	139.122	153.472	-23.975	42.049	-43.314	30.557	-4.295	166.839	44.483	-172.704	-74.261	170.308
8	A	8	U U	27.930	-138.622	-162.362	147.797	-27.578	42.721	-41.236	26.186	0.753	160.530	43.737	170.780	-89.103	-178.780
9	A	9	C C	14.533	-100.460	-142.464	115.688	-38.274	34.049	-17.800	-3.419	26.059	118.287	37.562	91.266	-140.318	73.980
10	A	10	C C	-104.942	157.105	-129.380	148.493	-19.568	36.256	-38.245	28.010	-5.501	169.266	38.927	-159.663	1.290	164.203
11	A	11	G G	-81.544	-86.543	-149.807	147.391	-19.316	35.462	-37.311	27.280	-5.187	169.004	38.009	161.832	-148.536	-123.961
12	A	12	C C	102.830	-169.643	-174.958	147.445	-24.472	40.063	-39.776	26.653	-1.533	163.462	41.493	179.044	-84.161	-174.064
13	A	13	C C	20.369	-123.772	-155.549	142.191	-24.795	37.642	-35.636	22.304	1.418	159.251	38.108	144.063		
14	A	14	A A	122.579	154.042	161.777	151.082	-10.431	29.934	-37.025	32.032	-13.727	-177.289	37.067	-171.022	-18.443	47.018
15	B	1	U u	....	....	-30.150	-89.321	-14.337	32.553	-37.461	30.265	-10.157	176.789	37.520	135.856	149.632	73.630
16	B	2	G G	49.832	-176.983	-47.430	-81.485	13.007	11.534	-30.058	38.364	-32.290	-142.057	38.114	123.003	151.421	47.830
17	B	3	G G	127.835	-113.124	-136.068	-90.938	-15.433	32.675	-36.613	28.786	-8.553	174.541	36.780	157.840	145.918	61.988
18	B	4	C C	62.413	178.813	-45.121	-83.364	-4.198	26.696	-37.783	36.446	-20.340	-167.447	38.708	146.012	148.632	72.414
19	B	5	G G	54.253	-177.329	-61.192	-139.575	26.364	-37.868	34.378	-20.216	-3.652	-24.253	37.706	111.871	167.008	108.029
20	B	6	C C	66.558	177.863	-41.409	-138.348	29.161	-39.497	34.419	-18.619	-6.415	-28.055	39.002	109.564	95.331	-156.438
21	B	7	U U	85.743	-114.863	-73.967	-87.212	20.752	0.869	-20.469	32.904	-33.730	-126.060	34.773	166.293	141.847	54.719
22	B	8	G G	-159.269	-149.195	-59.837	-84.186	-2.501	24.455	-35.857	35.489	-20.816	-165.106	37.103	-174.092	130.740	73.490
23	B	9	A A	57.641	-170.436	-38.110	-87.955	-13.283	32.510	-38.309	31.737	-11.737	178.873	38.317	160.226	117.589	81.954
24	B	10	U U	-2.720	149.771	-50.037	-131.620	30.899	-37.406	29.594	-12.757	-11.191	-36.232	36.688	138.811	81.007	-66.509
25	B	11	G G	145.610	166.222	-57.092	-97.247	-18.159	31.452	-32.103	22.684	-3.043	166.410	33.027	100.076	141.106	-90.807
26	B	12	A A	76.968	-161.162	-51.653	-88.391	-12.705	31.643	-37.555	31.352	-11.858	179.436	37.557	178.835	134.905	55.653
27	B	13	G G	70.783	-173.665	-55.989	-83.013	-5.360	27.907	-38.601	36.579	-19.683	-169.046	39.317	172.823	157.768	68.932
28	B	14	G G	60.248	170.333	-43.826	-80.264	2.680	21.905	-36.714	39.282	-26.401	-157.650	39.696	164.737	153.443	69.897
29	B	15	C C	82.818	-170.541	-71.076	-82.501	-0.045	23.293	-36.361	37.376	-23.509	-161.453	38.353	158.252	158.969	70.718
30	B	16	C C	70.138	166.520	-40.875	-85.044	-7.466	28.708	-37.865	34.639	-17.179	-172.354	38.205	145.770	151.035	61.549
31	B	17	G G	67.505	178.460	-46.256	-83.646	-4.475	26.740	-37.580	36.084	-19.936	-167.896	38.435	158.754	143.235	61.884
32	B	18	A A	-144.010	-151.773	-66.005	-88.061	-9.317	28.788	-36.221	31.961	-14.362	-175.784	36.319	168.074	142.551	62.504
33	B	19	A A	77.095	-162.934	-67.330	-88.760	-11.212	30.081	-36.448	31.098	-12.641	-178.751	36.456	163.753	153.127	59.288
34	B	20	A A	69.472	175.343	-46.558	-89.937	-13.350	31.391	-36.513	29.927	-10.569	177.836	36.539	147.238	126.420	86.797
35	B	21	G G	-112.427	114.230	157.866	-89.034	-13.496	32.092	-37.479	30.803	-11.011	178.126	37.499	-179.726	145.904	63.339
36	B	22	G G	63.018	175.722	-47.778	-82.389	-0.025	23.322	-36.405	37.435	-23.568	-161.423	38.407	168.556	154.708	64.725
37	B	23	C C	64.855	177.572	-46.466	-82.788	0.437	22.613	-35.729	37.029	-23.579	-160.736	37.848	150.547	155.438	70.799
38	B	24	C C	-105.567	157.954	132.617	-85.112	-5.394	26.873	-36.960	34.952	-18.654	-169.375	37.605	165.713	146.760	52.047
39	B	25	G G	72.267	-172.202	-58.431	-84.484	-2.572	24.339	-35.582	35.144	-20.544	-165.277	36.790	164.749	177.474	102.057
40	B	26	A A	55.182	163.830	-47.493	-89.910	-12.200	30.430	-36.082	30.141	-11.418	179.448	36.084	153.797	143.530	63.663
41	B	27	A A	62.127	-170.488	-47.747	-87.105	-10.480	30.376	-37.633	32.691	-14.083	-177.075	37.682	150.866	147.901	71.223
42	B	28	A A	67.271	-158.578	-70.648	-85.386	-7.201	28.307	-37.472	34.391	-17.194	-172.064	37.834	145.769	141.252	72.587
43	B	29	C C	77.089	-173.161	-56.942	-83.761	-6.833	28.964	-38.855	36.005	-18.412	-171.150	39.323	148.089	157.835	62.014
44	B	30	U U	68.637	170.888	-44.821	-81.557	2.658	21.213	-35.629	38.171	-25.698	-157.547	38.552	137.465	160.413	71.078
45	B	31	U U	69.745	-178.743	-54.363	-83.666	-1.426	23.835	-35.875	36.106	-21.871	-163.495	37.417	133.287	156.296	69.316
46	B	32	G G	79.973	-165.465	-70.702	-82.264	2.733	20.766	-34.963	37.546	-25.366	-157.360	37.882	152.883	157.461	61.872
47	B	33	A A	64.325	175.082	-46.354	-82.663	2.157	20.800	-34.467	36.747	-24.521	-158.109	37.146	139.233	....	....

Supplementary Figure 4. The values of all angles characteristic for nucleotide conformation for the D-target/L-ribozyme.

Supplementary Fig. 5

L-target / L-ribozyme

				alpha	beta	gamma	delta	v0	v1	v2	v3	v4	P	fi	chi	epsi	zeta
1	A	1	C c	....	....	-21.366	-90.226	-17.627	35.063	-38.302	29.235	-7.446	172.266	38.653	176.509	111.026	63.033
2	A	2	U U	46.787	-153.260	-54.614	-82.240	0.583	22.696	-36.023	37.437	-23.921	-160.507	38.213	160.081	141.569	58.962
3	A	3	U U	68.174	-176.487	-47.443	-86.061	-9.744	30.318	-38.280	33.747	-15.188	-175.693	38.389	156.899	154.743	64.293
4	A	4	C C	63.419	168.774	-35.807	-83.824	-6.584	28.629	-38.614	35.899	-18.514	-170.822	39.114	152.727	154.947	77.075
5	A	5	A A	56.952	179.178	-39.213	-85.106	-8.801	29.940	-38.553	34.581	-16.317	-174.150	38.755	148.829	154.722	71.277
6	A	6	A A	64.714	-169.374	-58.132	-82.239	-1.740	24.926	-37.312	37.383	-22.470	-163.880	38.839	160.765	155.226	74.293
7	A	7	G G	69.731	-179.326	-54.871	-83.841	-2.219	24.542	-36.234	36.022	-21.332	-164.659	37.573	155.639	156.622	59.029
8	A	8	U U	72.061	166.985	-42.143	-86.780	-3.167	23.695	-34.008	33.268	-19.007	-166.353	34.996	149.772	125.579	80.833
9	A	9	C C	49.994	-179.794	-44.694	-141.940	23.128	-35.618	33.805	-21.594	-0.744	-20.014	35.978	119.223	110.108	-87.206
10	A	10	C C	-78.990	141.368	75.633	-85.054	-7.454	28.578	-37.724	34.512	-17.094	-172.360	38.062	162.605	125.778	78.259
11	A	11	G G	80.927	-147.103	-83.618	-81.370	8.832	15.334	-32.154	38.145	-29.542	-148.272	37.803	-177.494	154.920	64.279
12	A	12	C C	74.374	163.385	-40.445	-84.984	-7.077	28.397	-37.737	34.751	-17.497	-171.782	38.129	149.326	124.839	82.385
13	A	13	C C	88.733	-115.061	-106.604	-86.786	-12.038	31.873	-38.565	32.679	-13.085	-179.105	38.569	177.337		
14	A	14	A A	74.069	162.507	-45.198	-86.712	-10.270	30.383	-37.838	33.021	-14.428	-176.660	37.902	162.724	70.740	30.014
15	B	1	U u	....	....	-57.917	-86.080	-6.279	27.184	-36.563	34.007	-17.538	-170.871	37.032	163.467	160.732	57.819
16	B	2	G G	69.475	162.123	-37.766	-81.471	-0.855	24.546	-37.548	38.127	-23.452	-162.616	39.345	154.141	133.265	79.203
17	B	3	G G	92.980	-121.417	-111.047	-86.319	-7.235	27.612	-36.362	33.277	-16.470	-172.416	36.683	179.582	145.097	67.763
18	B	4	C C	68.609	173.708	-44.451	-86.649	-10.354	30.554	-38.064	33.169	-14.438	-176.727	38.126	156.942	150.889	74.422
19	B	5	G G	64.480	172.070	-61.889	-146.574	18.233	-34.282	36.392	-26.971	5.696	-10.049	36.959	129.649	165.887	108.997
20	B	6	C C	67.396	175.810	-43.612	-138.742	30.431	-40.415	34.669	-18.156	-7.501	-29.428	39.805	119.443	88.370	-154.595
21	B	7	U U	80.029	-111.864	-76.116	-84.754	-1.832	23.626	-35.183	35.167	-21.026	-164.154	36.573	152.242	147.487	47.501
22	B	8	G G	-152.385	-149.229	-60.567	-85.197	-5.263	26.505	-36.476	34.529	-18.496	-169.278	37.124	-179.958	135.643	68.930
23	B	9	A A	59.584	-169.384	-42.132	-85.583	-13.886	34.393	-40.748	33.792	-12.663	179.167	40.752	160.222	114.470	69.838
24	B	10	U U	7.844	137.265	-41.712	-139.671	24.288	-36.031	33.499	-20.534	-2.147	-22.133	36.163	141.336	80.687	-59.751
25	B	11	G G	128.453	165.461	-52.811	-93.611	-17.874	33.483	-35.575	26.357	-5.514	169.909	36.134	76.689	155.810	-99.913
26	B	12	A A	48.652	-140.079	-38.378	-87.198	-10.988	30.667	-37.635	32.401	-13.583	-177.860	37.662	-177.297	127.770	64.205
27	B	13	G G	62.861	-166.158	-54.154	-82.511	-4.992	27.893	-38.933	37.129	-20.265	-168.440	39.739	173.510	158.088	71.101
28	B	14	G G	62.527	172.737	-49.002	-80.561	2.284	22.141	-36.749	39.107	-26.046	-158.174	39.587	167.878	149.165	71.564
29	B	15	C C	83.511	-170.006	-71.328	-81.767	-0.992	24.562	-37.456	37.915	-23.253	-162.832	39.202	165.707	158.311	71.753
30	B	16	C C	66.411	166.741	-38.710	-85.536	-8.909	29.894	-38.384	34.359	-16.092	-174.369	38.570	151.287	153.911	59.607
31	B	17	G G	69.482	175.912	-45.580	-83.599	-5.690	27.922	-38.303	36.122	-19.198	-169.567	38.947	155.190	142.321	63.266
32	B	18	A A	-144.611	-154.520	-62.997	-88.616	-10.229	29.227	-36.033	31.244	-13.336	-177.355	36.072	171.537	141.592	67.299
33	B	19	A A	73.744	-161.422	-66.845	-89.134	-11.486	30.159	-36.325	30.801	-12.281	-179.264	36.328	164.032	157.899	51.759
34	B	20	A A	78.586	164.728	-45.923	-91.301	-14.997	32.116	-36.084	28.543	-8.669	174.906	36.227	144.394	137.327	80.967
35	B	21	G G	-120.757	120.675	160.908	-88.132	-12.608	31.902	-38.009	31.870	-12.251	179.809	38.010	177.614	145.813	64.435
36	B	22	G G	66.079	178.400	-53.428	-81.092	3.145	20.995	-35.720	38.552	-26.245	-156.876	38.841	167.010	155.551	61.680
37	B	23	C C	69.058	175.907	-48.491	-82.190	2.432	20.969	-35.001	37.440	-25.101	-157.781	37.809	152.722	153.336	73.972
38	B	24	C C	-101.884	156.775	129.683	-84.721	-6.539	28.161	-37.891	35.195	-18.080	-170.950	38.369	164.723	145.073	53.673
39	B	25	G G	67.769	-163.185	-64.541	-83.999	-2.636	24.815	-36.308	35.847	-20.940	-165.290	37.538	168.405	167.313	108.742
40	B	26	A A	57.211	166.720	-46.860	-88.982	-11.980	30.670	-36.650	30.862	-12.008	-179.889	36.650	161.581	146.149	59.852
41	B	27	A A	67.364	-174.504	-48.661	-86.072	-11.404	31.763	-38.959	33.478	-13.997	-177.942	38.985	156.068	147.217	75.234
42	B	28	A A	61.972	-156.179	-62.698	-85.665	-7.444	28.391	-37.364	34.174	-16.911	-172.446	37.691	160.529	140.536	76.901
43	B	29	C C	64.925	179.689	-40.847	-85.806	-10.506	31.173	-38.862	33.903	-14.850	-176.615	38.930	155.144	157.121	68.728
44	B	30	U U	63.304	173.959	-41.453	-85.990	-8.513	29.272	-37.742	33.906	-16.078	-174.005	37.949	149.143	154.912	77.649
45	B	31	U U	60.871	-178.141	-45.900	-85.006	-7.255	28.461	-37.695	34.623	-17.292	-172.052	38.061	150.496	151.162	77.442
46	B	32	G G	73.614	-165.784	-65.777	-84.089	-3.736	25.845	-36.853	35.802	-20.215	-166.880	37.841	161.707	157.906	56.055
47	B	33	A A	73.534	160.870	-37.994	-84.918	-3.987	25.264	-35.690	34.500	-19.274	-167.415	36.568	150.788	....	....

Supplementary Figure 5. The values of all angles characteristic for nucleotide conformation for the L-target/L-ribozyme.

Supplementary Fig. 6

L-target / D-ribozyme

					alpha	beta	gamma	delta	v0	v1	v2	v3	v4	P	fi	chi	epsi	zeta
1	A	1	C	c	....	....	-164.222	-146.659	19.375	-35.116	36.601	-26.458	4.659	-11.723	37.381	144.241	-13.607	-142.497
2	A	2	U	U	78.887	94.873	153.391	-150.023	23.339	-40.503	41.712	-29.198	3.783	-13.517	42.900	-170.421	75.401	172.580
3	A	3	U	U	-8.817	117.388	-177.122	-146.878	31.210	-45.305	41.734	-24.706	-3.889	-23.443	45.489	166.375	76.543	-179.578
4	A	4	C	C	-18.954	139.762	154.618	-154.137	21.027	-40.115	43.093	-31.851	6.946	-9.563	43.701	178.880	27.506	179.805
5	A	5	A	A	35.403	97.865	156.265	-135.915	32.079	-40.331	33.142	-15.635	-10.124	-33.259	39.635	-147.522	96.868	126.533
6	A	6	A	A	-32.259	121.755	154.915	-131.336	27.558	-35.260	29.300	-14.385	-8.057	-32.065	34.574	-159.336	-52.774	-86.271
7	A	7	G	G	-91.145	-130.433	-139.122	-153.472	23.975	-42.049	43.314	-30.557	4.295	-13.161	44.483	172.704	74.261	-170.308
8	A	8	U	U	-27.930	138.622	162.362	-147.797	27.578	-42.721	41.236	-26.186	-0.753	-19.470	43.737	-170.780	89.103	178.780
9	A	9	C	C	-14.533	100.460	142.464	-115.688	38.274	-34.049	17.800	3.419	-26.059	-61.713	37.562	-91.266	140.318	-73.980
10	A	10	C	C	104.942	-157.105	129.380	-148.493	19.568	-36.256	38.245	-28.010	5.501	-10.734	38.927	159.663	-1.290	-164.203
11	A	11	G	G	81.544	86.543	149.807	-147.391	19.316	-35.462	37.311	-27.280	5.187	-10.996	38.009	-161.832	148.536	123.961
12	A	12	C	C	-102.830	169.643	174.958	-147.445	24.472	-40.063	39.776	-26.653	1.533	-16.538	41.493	-179.044	84.161	174.064
13	A	13	C	C	-20.369	123.772	155.549	-142.191	24.795	-37.642	35.636	-22.304	-1.418	-20.749	38.108	-144.063		
14	A	14	A	A	-122.579	-154.042	-161.777	-151.082	10.431	-29.934	37.025	-32.032	13.727	2.711	37.067	171.022	18.443	-47.018
15	B	1	U	u	....	....	30.150	89.321	14.337	-32.553	37.461	-30.265	10.157	-3.211	37.520	-135.856	-149.632	-73.630
16	B	2	G	G	-49.832	176.983	47.430	81.485	-13.007	-11.534	30.058	-38.364	32.290	37.943	38.114	-123.003	-151.421	-47.830
17	B	3	G	G	-127.835	113.124	136.068	90.938	15.433	-32.675	36.613	-28.786	8.553	-5.459	36.780	-157.840	-145.918	-61.988
18	B	4	C	C	-62.413	-178.813	45.121	83.364	4.198	-26.696	37.783	-36.446	20.340	12.553	38.708	-146.012	-148.632	-72.414
19	B	5	G	G	-54.253	177.329	61.192	139.575	-26.364	37.868	-34.378	20.216	3.652	155.747	37.706	-111.871	-167.008	-108.029
20	B	6	C	C	-66.558	-177.863	41.409	138.348	-29.161	39.497	-34.419	18.619	6.415	151.945	39.002	-109.564	-95.331	156.438
21	B	7	U	U	-85.743	114.863	73.967	87.212	-20.752	-0.869	20.469	-32.904	33.730	53.940	34.773	-166.293	-141.847	-54.719
22	B	8	G	G	159.269	149.195	59.837	84.186	2.501	-24.455	35.857	-35.489	20.816	14.894	37.103	174.092	-130.740	-73.490
23	B	9	A	A	-57.641	170.436	38.110	87.955	13.283	-32.510	38.309	-31.737	11.737	-1.127	38.317	-160.226	-117.589	-81.954
24	B	10	U	U	2.720	-149.771	50.037	131.620	-30.899	37.406	-29.594	12.757	11.191	143.768	36.688	-138.811	-81.007	66.509
25	B	11	G	G	-145.610	-166.222	57.092	97.247	18.159	-31.452	32.103	-22.684	3.043	-13.590	33.027	-100.076	-141.106	90.807
26	B	12	A	A	-76.968	161.162	51.653	88.391	12.705	-31.643	37.555	-31.352	11.858	-0.564	37.557	-178.835	-134.905	-55.653
27	B	13	G	G	-70.783	173.665	55.989	83.013	5.360	-27.907	38.601	-36.579	19.683	10.954	39.317	-172.823	-157.768	-68.932
28	B	14	G	G	-60.248	-170.333	43.826	80.264	-2.680	-21.905	36.714	-39.282	26.401	22.350	39.696	-164.737	-153.443	-69.897
29	B	15	C	C	-82.818	170.541	71.076	82.501	0.045	-23.293	36.361	-37.376	23.509	18.547	38.353	-158.252	-158.969	-70.718
30	B	16	C	C	-70.138	-166.520	40.875	85.044	7.466	-28.708	37.865	-34.639	17.179	7.646	38.205	-145.770	-151.035	-61.549
31	B	17	G	G	-67.505	-178.460	46.256	83.646	4.475	-26.740	37.580	-36.084	19.936	12.104	38.435	-158.754	-143.235	-61.884
32	B	18	A	A	144.010	151.773	66.005	88.061	9.317	-28.788	36.221	-31.961	14.362	4.216	36.319	-168.074	-142.551	-62.504
33	B	19	A	A	-77.095	162.934	67.330	88.760	11.212	-30.081	36.448	-31.098	12.641	1.249	36.456	-163.753	-153.127	-59.288
34	B	20	A	A	-69.472	-175.343	46.558	89.937	13.350	-31.391	36.513	-29.927	10.569	-2.164	36.539	-147.238	-126.420	-86.797
35	B	21	G	G	112.427	-114.230	-157.866	89.034	13.496	-32.092	37.479	-30.803	11.011	-1.874	37.499	179.726	-145.904	-63.339
36	B	22	G	G	-63.018	-175.722	47.778	82.389	0.025	-23.322	36.405	-37.435	23.568	18.577	38.407	-168.556	-154.708	-64.725
37	B	23	C	C	-64.855	-177.572	46.466	82.788	-0.437	-22.613	35.729	-37.029	23.579	19.264	37.848	-150.547	-155.438	-70.799
38	B	24	C	C	105.567	-157.954	-132.617	85.112	5.394	-26.873	36.960	-34.952	18.654	10.625	37.605	-165.713	-146.760	-52.047
39	B	25	G	G	-72.267	172.202	58.431	84.484	2.572	-24.339	35.582	-35.144	20.544	14.723	36.790	-164.749	-177.474	-102.057
40	B	26	A	A	-55.182	-163.830	47.493	89.910	12.200	-30.430	36.082	-30.141	11.418	-0.552	36.084	-153.797	-143.530	-63.663
41	B	27	A	A	-62.127	170.488	47.747	87.105	10.480	-30.376	37.633	-32.691	14.083	2.925	37.682	-150.866	-147.901	-71.223
42	B	28	A	A	-67.271	158.578	70.648	85.386	7.201	-28.307	37.472	-34.391	17.194	7.936	37.834	-145.769	-141.252	-72.587
43	B	29	C	C	-77.089	173.161	56.942	83.761	6.833	-28.964	38.855	-36.005	18.412	8.850	39.323	-148.089	-157.835	-62.014
44	B	30	U	U	-68.637	-170.888	44.821	81.557	-2.658	-21.213	35.629	-38.171	25.698	22.453	38.552	-137.465	-160.413	-71.078
45	B	31	U	U	-69.745	178.743	54.363	83.666	1.426	-23.835	35.875	-36.106	21.871	16.505	37.417	-133.287	-156.296	-69.316
46	B	32	G	G	-79.973	165.465	70.702	82.264	-2.733	-20.766	34.963	-37.546	25.366	22.640	37.882	-152.883	-157.461	-61.872
47	B	33	A	A	-64.325	-175.082	46.354	82.663	-2.157	-20.800	34.467	-36.747	24.521	21.891	37.146	-139.233	....	....

Supplementary Figure 6. The values of all angles characteristic for nucleotide conformation for the L-target/D-ribozyme.

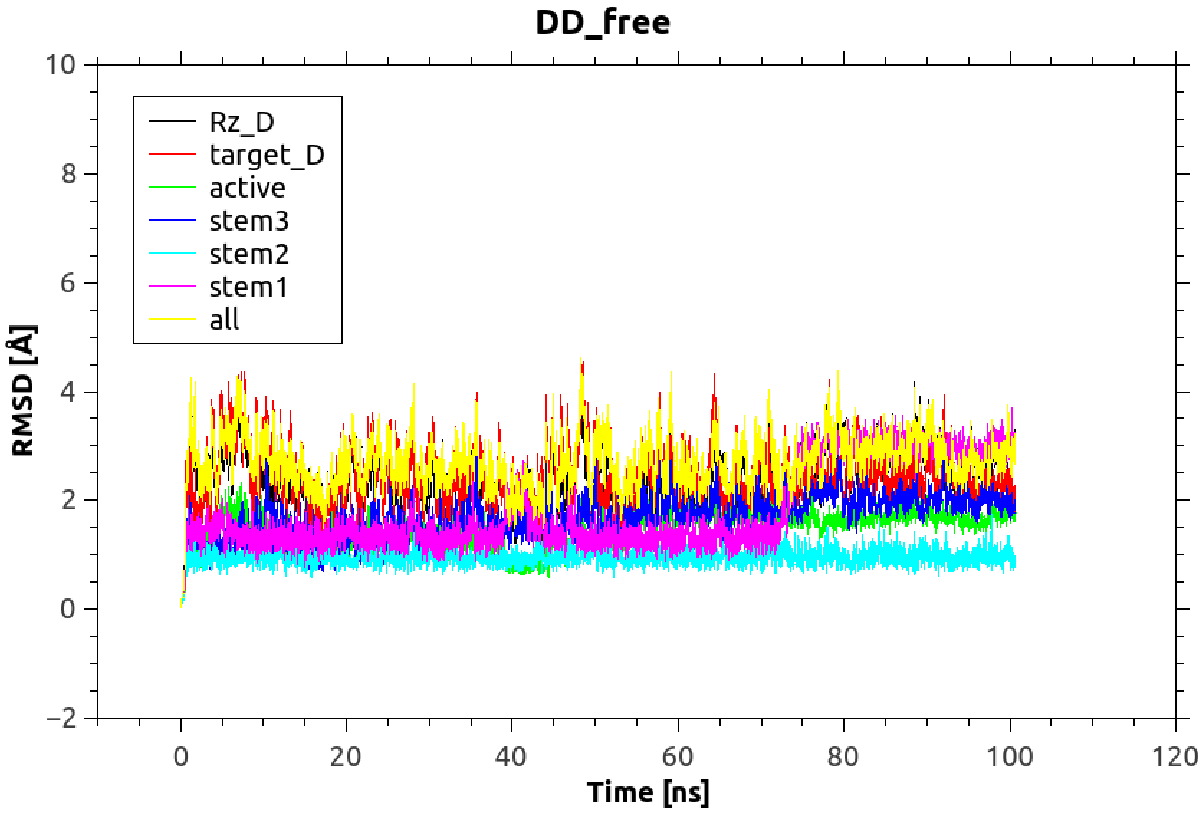
DD_free
RMSD [Å]
Time [ns]
Rz_D
target_D
active
stem3
stem2
stem1
all
10
8
6
4
2
0
−2
0
20
40
60
80
100
120

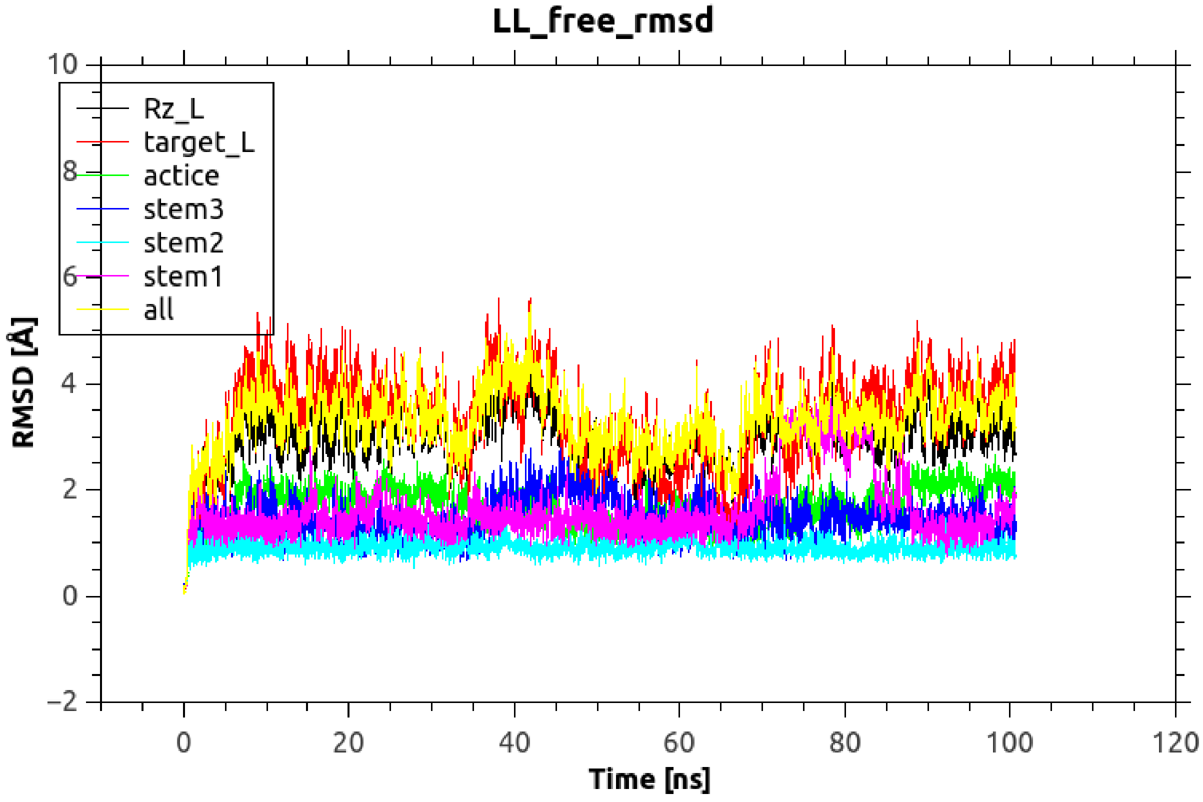
LL_free_rmsd
RMSD [Å]
Time [ns]
Rz_L
target_L
actice
stem3
stem2
stem1
all
10
8
6
4
2
0
−2
0
20
40
60
80
100
120

Supplementary Figure 7. RMSD for the whole homochiral ribozyme complexes with RNA (D/D or L/L) and their structural domains calculated without any restrains (free).

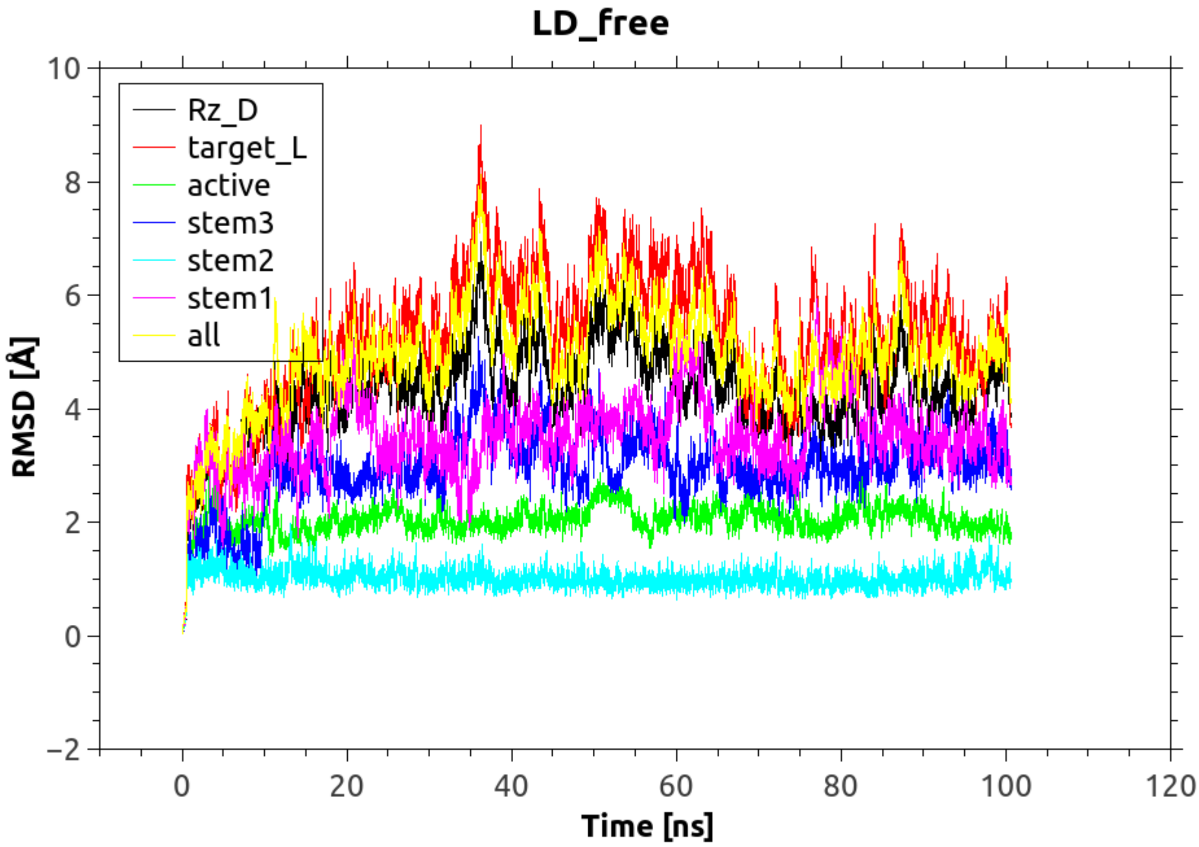
LD_free
Rz_D
target_L
active
stem3
stem2
stem1
all
RMSD [Å]
Time [ns]

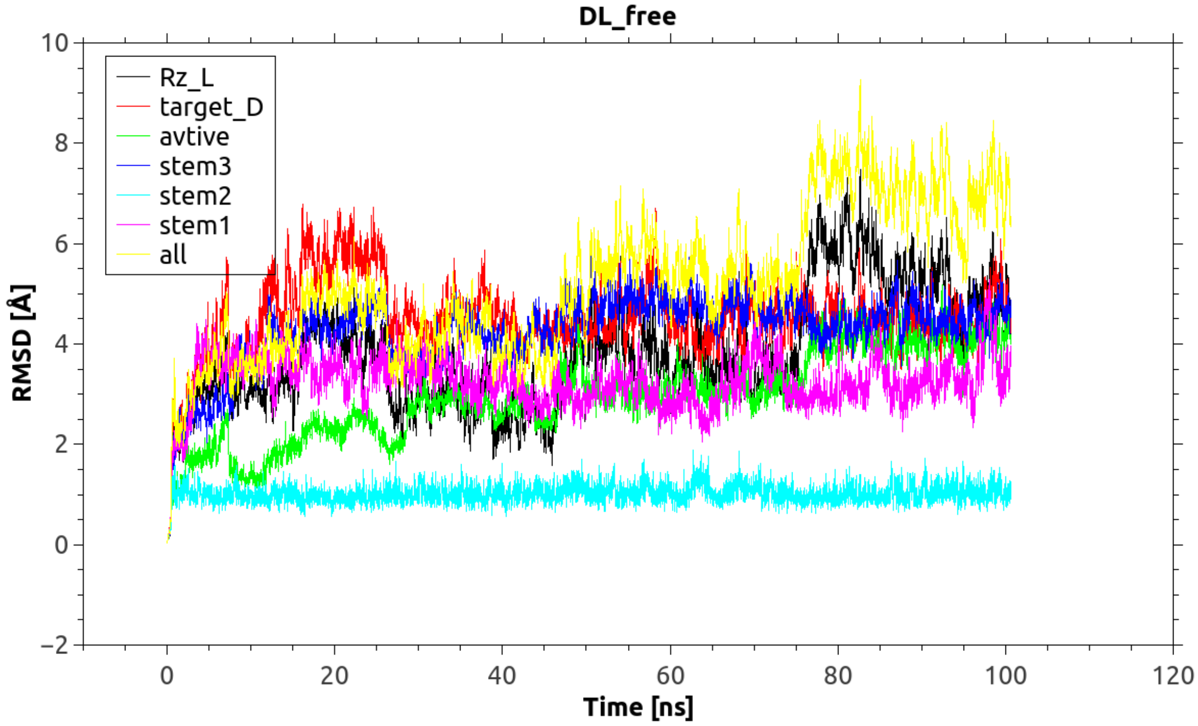
DL_free
Rz_L
target_D
avtive
stem3
stem2
stem1
all
RMSD [Å]
Time [ns]

Supplementary Figure 8. RMSD for the whole ribozyme heterochiral complexes with substrate (LD or DL) and their structural domains calculated without any restrains.

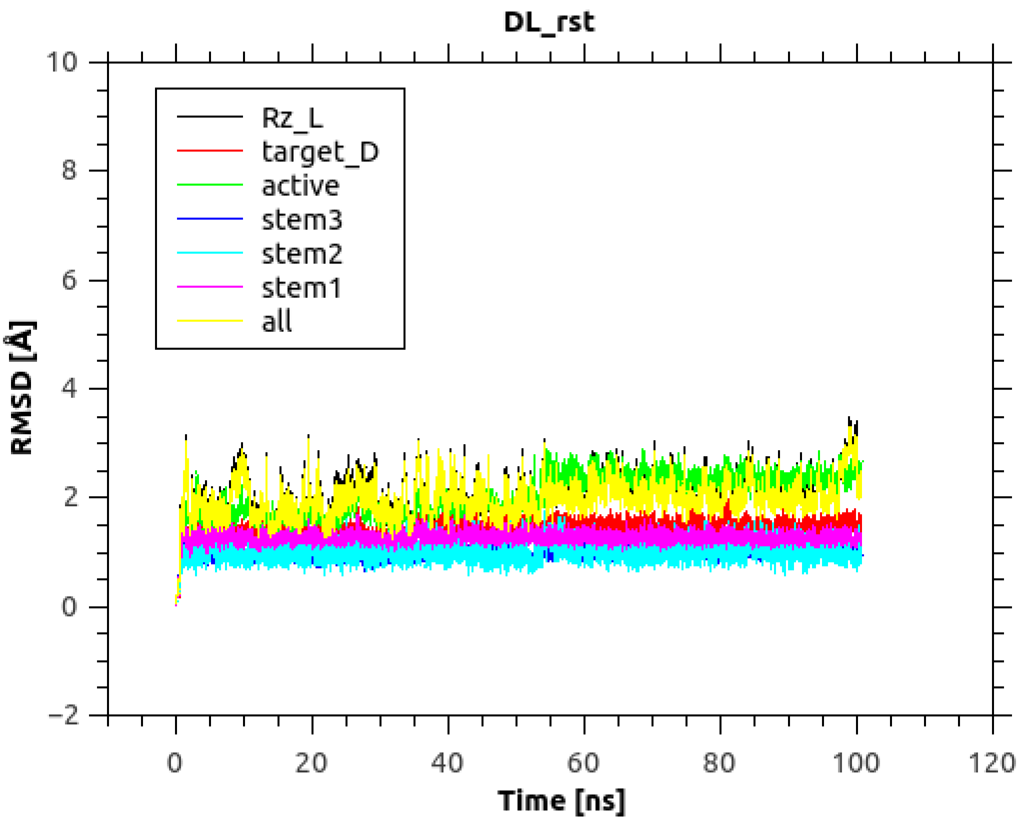

DL_rst
Rz_L
target_D
active
stem3
stem2
stem1
all
RMSD [Å]
Time [ns]

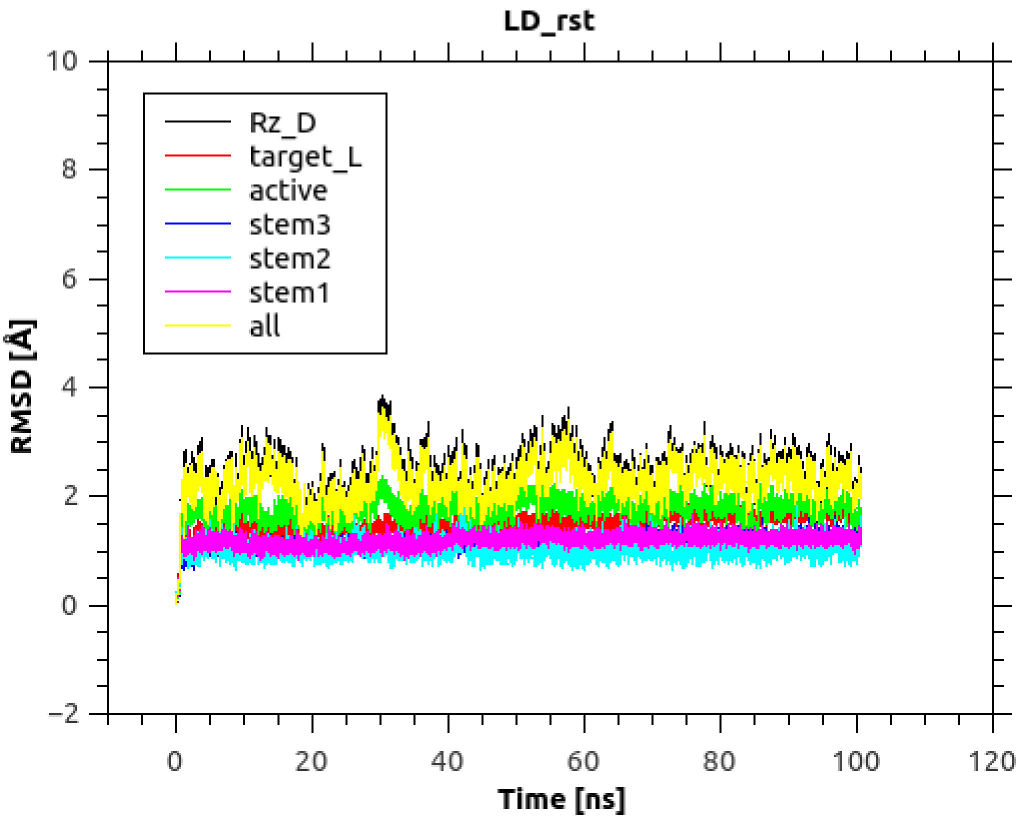

LD_rst
Rz_D
target_L
active
stem3
stem2
stem1
all
RMSD [Å]
Time [ns]

Supplementary Figure 9. RMSD for the whole heterochiral ribozyme complex with substrate (D/L or L/D) and their structural domains calculated with positional restrains (heavy atoms).
